# Causal association between cataract and psychiatric disorders: A bidirectional 2-sample Mendelian randomization study

**DOI:** 10.1097/MD.0000000000050075

**Published:** 2026-08-07

**Authors:** Xiaoling Su, Guoqiang Tian

**Affiliations:** a Department of Psychiatry, Shengzhou Traditional Chinese Medicine Hospital, Shengzhou, Zhejiang, China; b Department of Psychiatry, Seventh People’s Hospital of Shaoxing, Shaoxing, Zhejiang, China.

**Keywords:** cataract, causal association, genetic analyses, Mendelian randomization, psychiatric disorders

## Abstract

This study aimed to investigate the genetic causality between cataract (senile and other types) and psychiatric disorders using Mendelian randomization (MR). We performed a bidirectional 2-sample MR analysis using genome-wide association studies summary data. Primary analysis employed inverse-variance weighted, MR-Egger, weighted median, and weighted mode methods. Sensitivity analyses included Cochran *Q*, MR-pleiotropy residual sum and outlier, MR-Egger intercept, and leave-one-out tests to assess heterogeneity and pleiotropy. Forward MR analysis revealed a suggestive causal effect of other cataract on anxiety (inverse-variance weighted odds ratio [OR] = 0.99, 95% confidence interval: 0.98–1.00, *P* = .043), though unsupported by other methods. MR-Egger and weighted median suggested potential associations between other cataract and bipolar disorder (*P* < .05). No significant links were found between senile cataract and psychiatric disorders. Reverse MR analysis showed no consistent causal effects, except for anxiety disorder on other cataract (MR-Egger OR = 8.04, *P* = .040; weighted median OR = 2.99, *P* = .047). Sensitivity analyses confirmed robustness despite heterogeneity. This study provides evidence of a potential causal relationship between other cataract and anxiety, though findings were method-dependent. No significant associations were observed for senile cataract or most psychiatric disorders.

## 1. Introduction

Psychiatric disorders have emerged as a significant public health concern, characterized by high morbidity and mortality rates.^[1]^ It is estimated that the global burden of psychiatric disorders accounts for nearly 21.2% to 32.4% of years lived with disability.^[2]^ Despite advancements, the management of psychiatric disorders in clinical settings remains challenging, often focusing on symptomatic relief rather than addressing the underlying etiologies.^[3]^ Consequently, there is an urgent need for a deeper understanding of the pathophysiology and potential risk factors of psychiatric disorders to develop innovative prevention and intervention strategies. The etiology of psychiatric disorders remains largely enigmatic. Emerging evidence suggests that the causes are multifactorial, encompassing environmental and genetic factors, as well as parental psychopathological conditions.^[4]^ Recently, there has been a surge of interest in the relationship between ocular diseases, particularly cataract, and psychiatric disorders.

Cataract, characterized by the progressive opacification of the lens within the eye, is a prevalent cause of vision impairment and loss.^[5]^ Mental illness is more common in people with cataracts. For instance, previous observational studies have indicated that children and adolescents with 5 severe structural eye diseases, including cataract, are at a higher risk of psychiatric disorders compared to their peers without these conditions.^[6]^ In another study, children with cataract were found to have nearly double the incidence of psychiatric disorders, with a fourfold increase in the risk of anxiety disorders, especially if diagnosed before age 3.^[7]^ Some cross-sectional studies have observed higher rates of cataract, glaucoma, and dry eye syndrome in veterans with serious psychiatric disorders compared to those without.^[8]^ These studies suggest a correlation between cataract and psychiatric disorders, although the associations may be influenced by the inherent biases and confounding factors. Furthermore, the debate continues on whether cataract is a cause or a downstream effect of psychiatric disorders, and thus, the potential causal relationships warrant further investigation.

Mendelian randomization (MR), a genetic epidemiological approach, serves as a valuable tool for assessing the causal role of cataract in mental illness. Utilizing genetic variants such as single-nucleotide polymorphisms (SNPs) as instrumental variables (IVs) for modifiable disease risk factors or exposures, MR can enhance the causal inference on an exposure-outcome association.^[9,10]^ Genetic variants are randomly allocated during gamete formation, making them less susceptible to confounding factors. Additionally, since genotypes are immutable to the development of disease, MR minimizes the impact of confounding factors and reverse causation.^[11,12]^ To elucidate the association between cataract and psychiatric disorders and to evaluate the direction of such associations, we conducted a 2-sample bidirectional MR study using genome-wide association studies (GWASs).

## 2. Methods

### 2.1. Study design

**Figure 1.**
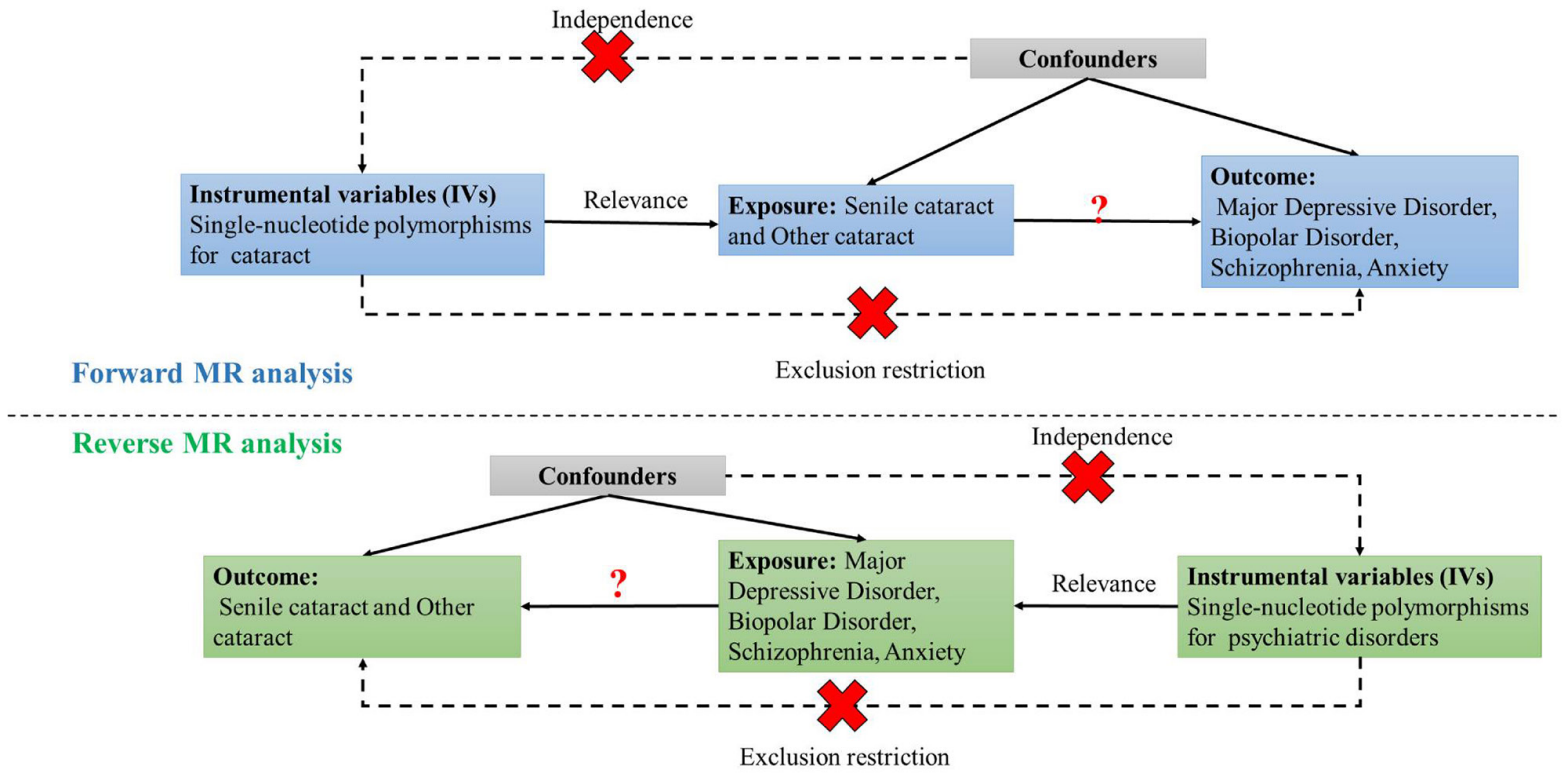
Description of the study design in this bidirectional MR study based on 3 core assumptions. Relevance: the genetic variants are associated with the exposure; independence: the genetic variants are not associated with confounders linked to the chosen exposure and outcome; exclusion restriction: the genetic variants influence the outcome only through the exposures and not via other biological pathways. MR = Mendelian randomization, SNPs = single-nucleotide polymorphisms.

A schematic overview of the study design is depicted in Figure 1. We conducted a bidirectional 2-sample MR analysis using GWAS summary statistics to explore the causal association between cataract and psychiatric disorders. The forward MR analysis considered cataract as the exposure and psychiatric disorders as the outcome, while the reverse MR analysis considered psychiatric disorders as the exposure and cataract as the outcome. The core MR assumptions are as follows: relevance – the genetic variants are associated with the exposure; independence – the genetic variants are not associated with confounders linked to the chosen exposure and outcome; exclusion restriction – the genetic variants influence the outcome only through the exposures and not via other biological pathways.^[11]^

### 2.2. Data sources

The GWAS summary data for cataract, including age-related cataract (65,235 cases and 341,546 controls) and other cataracts (19,401 cases and 341,546 controls), were sourced from the FinnGen consortium.^[13]^ The disease phenotype is defined from the health registry based on international classification of diseases 8 (ICD-8), ICD-9 and ICD-10 codes. The FinnGen study’s key strength lies in its rigorous bioinformatic pipeline. Genotypes were imputed using a population-specific SISu reference panel, which contains high-coverage whole-genome sequence data from thousands of Finns. The genome-wide association analyses were performed using a mixed model, SAIGE (Scalable and Accurate Implementation of GEneralized mixed model) – an open-source academic R package – to effectively control for cryptic relatedness and population structure, ensuring robust results.^[14]^ Psychiatric disorder data, including anxiety, were extracted from the Medical Research Council Integrative Epidemiology Unit database, major depressive disorder from research conducted by Wray et al,^[15]^ bipolar disorder from the study by Stahl et al,^[16]^ and schizophrenia from the work of Trubetskoy et al.^[17]^ For GWAS data on schizophrenia and major depressive disorder, quality control was performed according to the standards developed by the psychiatric genomics consortium.^[18]^ All GWAS summary statistics were from European-ancestry cohorts and underwent stringent quality control. Importantly, the exposure and outcome datasets were independent with no sample overlap. Comprehensive data information is provided in Table S1, Supplementary Digital Content 1. As this research utilizes only publicly accessible summary data, ethical review or consent is not required.

### 2.3. Instrumental variable selection

The selection of IVs in this study adhered to the following criteria. Firstly, SNPs significantly associated with major depressive disorder, bipolar disorder, schizophrenia, senile cataract, and other cataract were identified with a *P* value threshold of <5 × 10^−8^; for Anxiety, due to the limited number of SNPs identified with *P* < 5 × 10^−8^, the threshold was adjusted to *P* < 5 × 10^−6^.^[19]^ Secondly, SNPs with a minor allele frequency exceeding 0.01 were selected to ensure that the genetic variants are common in the population.^[19]^ Thirdly, to counteract the influence of linkage disequilibrium (LD) among SNPs, a rigorous LD exclusion criterion was applied (*R*^2^ < 0.001 within a window size of 10,000 kb), thereby ensuring the autonomy of the chosen IVs.^[20]^ Fourthly, in scenarios where the initially selected IV was not represented in the outcome’s summary dataset, a proxy SNP in high LD (*R*^2^ > 0.8) with the selected IV was identified to effectively encapsulate the genetic impact of the exposure.^[19]^ Fifthly, to assess the strength of the selected IVs, we calculated the *F* statistic for each SNP using the formula: *F* = *R*^2^ × (N − 2)/(1 − *R*^2^), where *R*^2^ denotes the proportion of the exposure’s variability attributed to the SNP within the IV.^[21,22]^ SNPs with an *F* statistic below 10 were considered weak IVs and subsequently discarded.

### 2.4. Mendelian randomization analysis

The analysis predominantly utilized the inverse-variance weighted (IVW) method to evaluate the causal nexus between exposure and outcome, quantifying the odds ratio (OR) alongside the 95% confidence interval (CI).^[23]^ The IVW method, which apportions the effect size by the inverse variance of each SNP, stands as the cornerstone for elucidating MR findings. Complementing the IVW method, the MR-Egger regression, weighted median, and weighted mode methods were engaged to substantiate the robustness of our findings. The MR-Egger method, which incorporates an intercept, facilitates precise estimation of the causal impact even amidst pleiotropy bias.^[24]^ The weighted median method, predicated on the assumption that half of the instrumental variables are valid, dissects the causal link between exposure and outcome.^[25]^ The analyses were executed using R version 4.3.2 (R Development Core Team, R Foundation for Statistical Computing), harnessing the “TwoSampleMR” package, with findings delineated in forest plots, scatter plots, and funnel plots.

### 2.5. Sensitivity analysis

The heterogeneity among the IVs was gauged using Cochran *Q* test.^[26]^ A *P* value >.05 denoted low heterogeneity, inferring that the valuations among the instrumental variables exhibited random variability with negligible ramifications on the IVW results. To address the genetic pleiotropy’s impact on the causal effect’s estimation, the MR-Egger regression method was deployed to scrutinize the presence of horizontal pleiotropy. An intercept term in the MR-Egger regression approximating zero or deemed statistically insignificant indicates no pleiotropy.^[24]^ Moreover, the MR pleiotropy residual sum and outlier (MR-PRESSO) method was applied to detect and exclude potential outliers (SNPs with *P* < .05), followed by a reestimation of the causal association post-their exclusion, thus rectifying for horizontal pleiotropy.^[27]^ A leave-one-out analysis was conducted to discern whether any singular SNP wielded influence over the observed causal relationship.^[24]^

## 3. Results

### 3.1. Instrumental variable selection

When psychiatric disorders were considered as the outcome, the study identified 42 and 16 IVs associated with senile cataract and other cataract, respectively. Notably, 9 SNPs did not match any information in the summary data (Table S2, Supplementary Digital Content 2). Conversely, when cataract was the outcome, 5, 16, 158, and 17 IVs related to major depressive disorder, bipolar disorder, schizophrenia, and anxiety were identified, respectively, with 5 SNPs not found in the summary data (Table S3, Supplementary Digital Content 3). All selected instrumental variables were sufficiently strong, with *F* statistics for each SNP substantially exceeding the conventional threshold of 10, which minimizes the risk of weak instrument bias.

### 3.2. The causal effect of cataract on psychiatric disorders in the forward MR analysis

**Figure 2.**
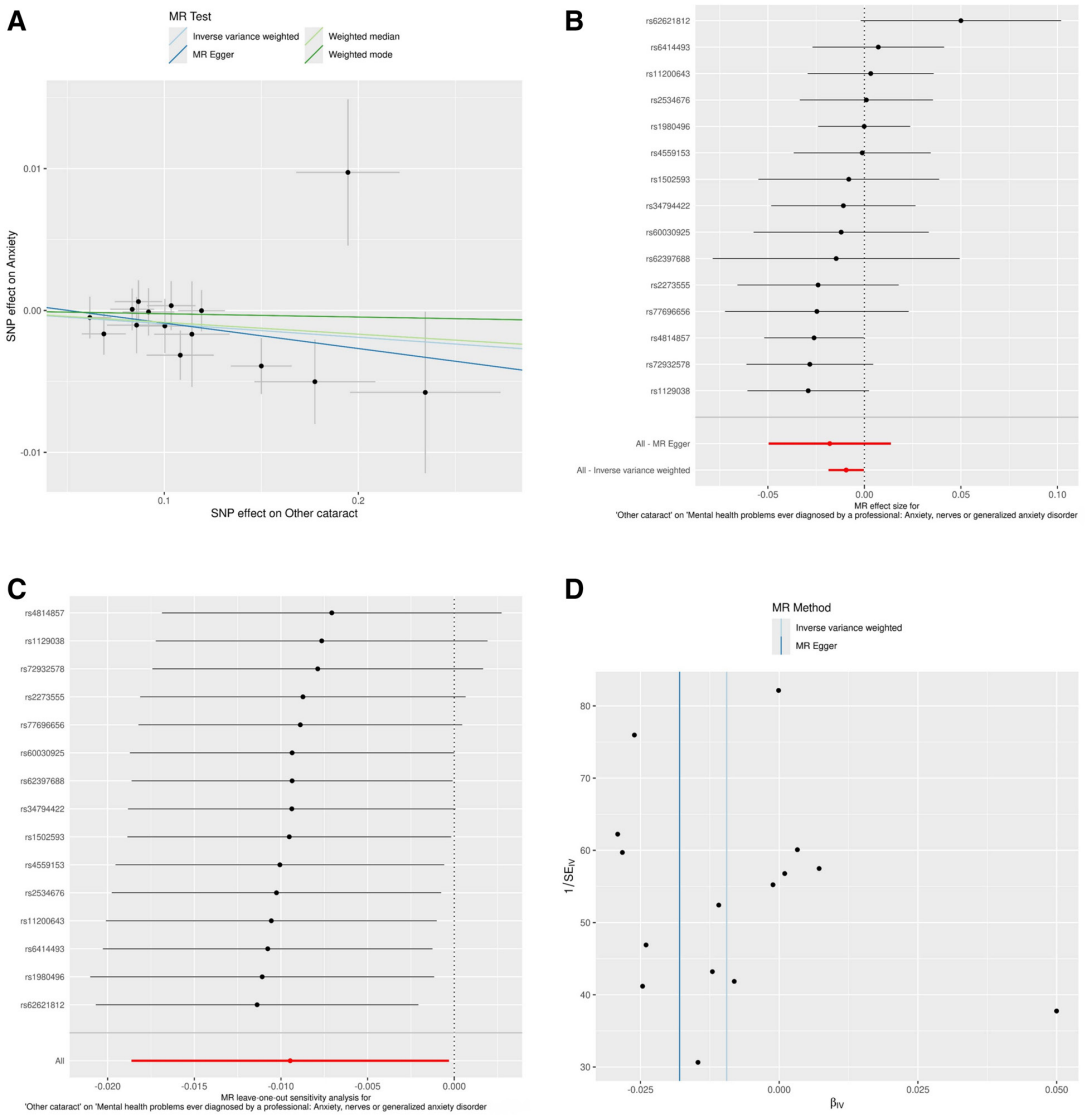
Causal effect of other cataracts on anxiety in the forward MR analysis. (A) Scatter plot of the association between other cataracts and anxiety. IVW, MR-Egger, weighted median, and weighted mode methods were applied. (B) A forest plot was used to show the MR estimate and 95% CI values (gray line segment) for each SNP, which also shows the IVW and MR-Egger results at the bottom. (C) Leave-one-out analysis to evaluate whether any single instrumental variable was driving the causal effect of other cataracts on anxiety. (D) A funnel plot was applied to detect whether the observed association was accompanied by obvious heterogeneity. CI = confidence interval, IVW = inverse-variance weighted, MR = Mendelian randomization, SNPs = single-nucleotide polymorphisms.

In the forward analysis, as shown in Table 1 and Figure 2A and B, the association between other cataract and anxiety was statistically significant by the IVW method (OR = 0.99, 95% CI: 0.98–1.00, *P* = .043), suggesting a reduced risk of anxiety with other cataract. However, this association was not corroborated by the MR-Egger (OR = 0.98, 95% CI: 0.95–1.01, *P* = .287), weighted median (OR = 0.99, 95% CI: 0.98–1.00, *P* = .196), or weighted mode (OR = 1.00, 95% CI: 0.98–1.02, *P* = .825) methods. The consistency of the association between other cataract and anxiety was further confirmed by leave-one-out sensitivity analysis, where the exclusion of any individual SNP did not alter the observed relationship, as shown in Figure 2C, reinforcing the reliability of the findings. The funnel plot (Fig. 2D; Fig. S1, Supplementary Digital Content 4) displayed symmetry on either side of the effect line, suggesting the research is unbiased.

**Table 1:** Genetically predicted association between cataracts and the risk of psychiatric disorders by MR analyses.

MR direction	Exposure	Outcome	Methods	NSNP	*P*	OR (95% CI)
Forward	Other cataract	Anxiety	IVW	15	.043	0.99 (0.98–1)
Forward	Other cataract	Anxiety	MR-Egger	15	.287	0.98 (0.95–1.01)
Forward	Other cataract	Anxiety	Weighted median	15	.196	0.99 (0.98–1)
Forward	Other cataract	Anxiety	Weighted mode	15	.825	1 (0.98–1.02)
Reverse	Anxiety disorder	Other cataracts	IVW	15	.075	2.06 (0.93–4.56)
Reverse	Anxiety disorder	Other cataracts	MR-Egger	15	.04	8.04 (1.34–48.3)
Reverse	Anxiety disorder	Other cataracts	Weighted median	15	.047	2.99 (1.02–8.79)
Reverse	Anxiety disorder	Other cataracts	Weighted mode	15	.121	4.32 (0.71–26.24)
Forward	Other cataract	Bipolar disorder	IVW	15	.876	1.01 (0.91–1.12)
Forward	Other cataract	Bipolar disorder	MR-Egger	15	.045	1.35 (1.04–1.76)
Forward	Other cataract	Bipolar disorder	Weighted median	15	.044	1.14 (1–1.29)
Forward	Other cataract	Bipolar disorder	Weighted mode	15	.156	1.14 (0.96–1.35)
Reverse	Bipolar disorder	Other cataracts	IVW	12	.691	0.98 (0.86–1.1)
Reverse	Bipolar disorder	Other cataracts	MR-Egger	12	.158	1.69 (0.86–3.33)
Reverse	Bipolar disorder	Other cataracts	Weighted median	12	.417	0.95 (0.84–1.08)
Reverse	Bipolar disorder	Other cataracts	Weighted mode	12	.607	1.07 (0.83–1.38)
Forward	Other cataract	Major depressive disorder	IVW	15	.834	1.01 (0.94–1.08)
Forward	Other cataract	Major depressive disorder	MR-Egger	15	.692	1.05 (0.84–1.3)
Forward	Other cataract	Major depressive disorder	Weighted median	15	.448	1.03 (0.95–1.12)
Forward	Other cataract	Major depressive disorder	Weighted mode	15	.809	1.01 (0.91–1.13)
Reverse	Major depressive disorder	Other cataracts	IVW	4	.196	1.21 (0.9–1.63)
Reverse	Major depressive disorder	Other cataracts	MR-Egger	4	.179	4.95 (1.06–23.07)
Reverse	Major depressive disorder	Other cataracts	Weighted median	4	.552	1.1 (0.81–1.5)
Reverse	Major depressive disorder	Other cataracts	Weighted mode	4	.886	1.03 (0.71–1.5)
Forward	Other cataract	Schizophrenia	IVW	12	.292	0.96 (0.9–1.03)
Forward	Other cataract	Schizophrenia	MR-Egger	12	.148	0.84 (0.67–1.04)
Forward	Other cataract	Schizophrenia	Weighted median	12	.541	0.97 (0.89–1.07)
Forward	Other cataract	Schizophrenia	Weighted mode	12	.893	0.99 (0.86–1.14)
Reverse	Schizophrenia	Other cataracts	IVW	150	.495	1.01 (0.98–1.05)
Reverse	Schizophrenia	Other cataracts	MR-Egger	150	.788	1.02 (0.88–1.18)
Reverse	Schizophrenia	Other cataracts	Weighted median	150	.876	1 (0.95–1.04)
Reverse	Schizophrenia	Other cataracts	Weighted mode	150	.48	0.96 (0.85–1.08)
Forward	Senile cataract	Anxiety	IVW	39	.65	1 (0.99–1.01)
Forward	Senile cataract	Anxiety	MR-Egger	39	.458	0.99 (0.96–1.02)
Forward	Senile cataract	Anxiety	Weighted median	39	.722	1 (0.99–1.01)
Forward	Senile cataract	Anxiety	Weighted mode	39	.925	1 (0.98–1.02)
Reverse	Anxiety disorder	Senile cataract	IVW	15	.108	1.56 (0.91–2.69)
Reverse	Anxiety disorder	Senile cataract	MR-Egger	15	.257	2.14 (0.61–7.57)
Reverse	Anxiety disorder	Senile cataract	Weighted median	15	.347	1.42 (0.68–2.96)
Reverse	Anxiety disorder	Senile cataract	Weighted mode	15	.651	1.32 (0.44–3.91)
Forward	Senile cataract	Bipolar disorder	IVW	37	.439	0.96 (0.86–1.07)
Forward	Senile cataract	Bipolar disorder	MR-Egger	37	.889	1.02 (0.77–1.35)
Forward	Senile cataract	Bipolar disorder	Weighted median	37	.233	0.92 (0.81–1.05)
Forward	Senile cataract	Bipolar disorder	Weighted mode	37	.383	0.92 (0.77–1.11)
Reverse	Bipolar disorder	Senile cataract	IVW	12	.833	0.99 (0.93–1.06)
Reverse	Bipolar disorder	Senile cataract	MR-Egger	12	.368	1.2 (0.82–1.77)
Reverse	Bipolar disorder	Senile cataract	Weighted median	12	.608	0.98 (0.91–1.06)
Reverse	Bipolar disorder	Senile cataract	Weighted mode	12	.925	0.99 (0.86–1.14)
Forward	Senile cataract	Major depressive disorder	IVW	37	.999	1 (0.95–1.06)
Forward	Senile cataract	Major depressive disorder	MR-Egger	37	.455	1.06 (0.91–1.23)
Forward	Senile cataract	Major depressive disorder	Weighted median	37	.22	1.05 (0.97–1.12)
Forward	Senile cataract	Major depressive disorder	Weighted mode	37	.325	1.05 (0.95–1.16)
Reverse	Major depressive disorder	Senile cataract	IVW	5	.413	1.06 (0.92–1.23)
Reverse	Major depressive disorder	Senile cataract	MR-Egger	5	.164	2.35 (0.94–5.86)
Reverse	Major depressive disorder	Senile cataract	Weighted median	5	.528	1.06 (0.89–1.26)
Reverse	Major depressive disorder	Senile cataract	Weighted mode	5	.628	1.07 (0.84–1.35)
Forward	Senile cataract	Schizophrenia	IVW	34	.961	1 (0.92–1.09)
Forward	Senile cataract	Schizophrenia	MR-Egger	34	.57	0.94 (0.75–1.17)
Forward	Senile cataract	Schizophrenia	Weighted median	34	.629	0.98 (0.9–1.06)
Forward	Senile cataract	Schizophrenia	Weighted mode	34	.749	0.98 (0.89–1.09)
Reverse	Schizophrenia	Senile cataract	IVW	150	.323	1.01 (0.99–1.04)
Reverse	Schizophrenia	Senile cataract	MR-Egger	150	.788	1.01 (0.91–1.12)
Reverse	Schizophrenia	Senile cataract	Weighted median	150	.239	1.02 (0.99–1.05)
Reverse	Schizophrenia	Senile cataract	Weighted mode	150	.389	0.96 (0.88–1.05)

CI = confidence interval, IVW = inverse-variance weighted, MR = Mendelian randomization, OR = odds ratio.

It is noteworthy that the MR-Egger (OR = 1.35, 95% CI: 1.04–1.76, *P* = .045) and weighted median (OR = 1.14, 95% CI: 1–1.29, *P* = .044) methods suggest a potential causal association between other cataract and bipolar disorder, as shown in Table 1. However, the IVW method (OR = 1.01, 95% CI: 0.91–1.12, *P* = .876) does not support this causal relationship. This study did not find a statistically significant association between other cataract and major depressive disorder, bipolar disorder, or schizophrenia (all *P* > .05, Table 1). Furthermore, no statistically significant association was observed between senile cataract and the 4 psychiatric disorders studied (all *P* > .05, Table 1). Notably, Cochran *Q* test within the IVW approach indicated heterogeneity in the relationship between other cataract and bipolar disorder (*Q* = 23.482, *P* = .015), other cataract and schizophrenia (*Q* = 193.851, *P* = .008), and senile cataract and schizophrenia (*Q* = 230.207, *P* < .001). Although the MR-Egger regression analysis suggested the absence of potential pleiotropy, the MR-PRESSO analysis detected horizontal pleiotropy for the association between senile cataract and major depressive disorder (global *P* = .045), senile cataract and bipolar disorder (global *P* < .001), and senile cataract and schizophrenia (global *P* < .001). Furthermore, 2 potential outlier SNPs (rs1231439, rs3094577) were identified for the association between senile cataract and schizophrenia. After removing these outliers, the causal estimate remained nonsignificant (OR = 0.97, 95% CI: 0.91–1.04, *P* = .416), confirming the robustness of our primary finding.

### 3.3. The causal effect of psychiatric disorders on cataract in the reverse MR analysis

**Figure 3.**
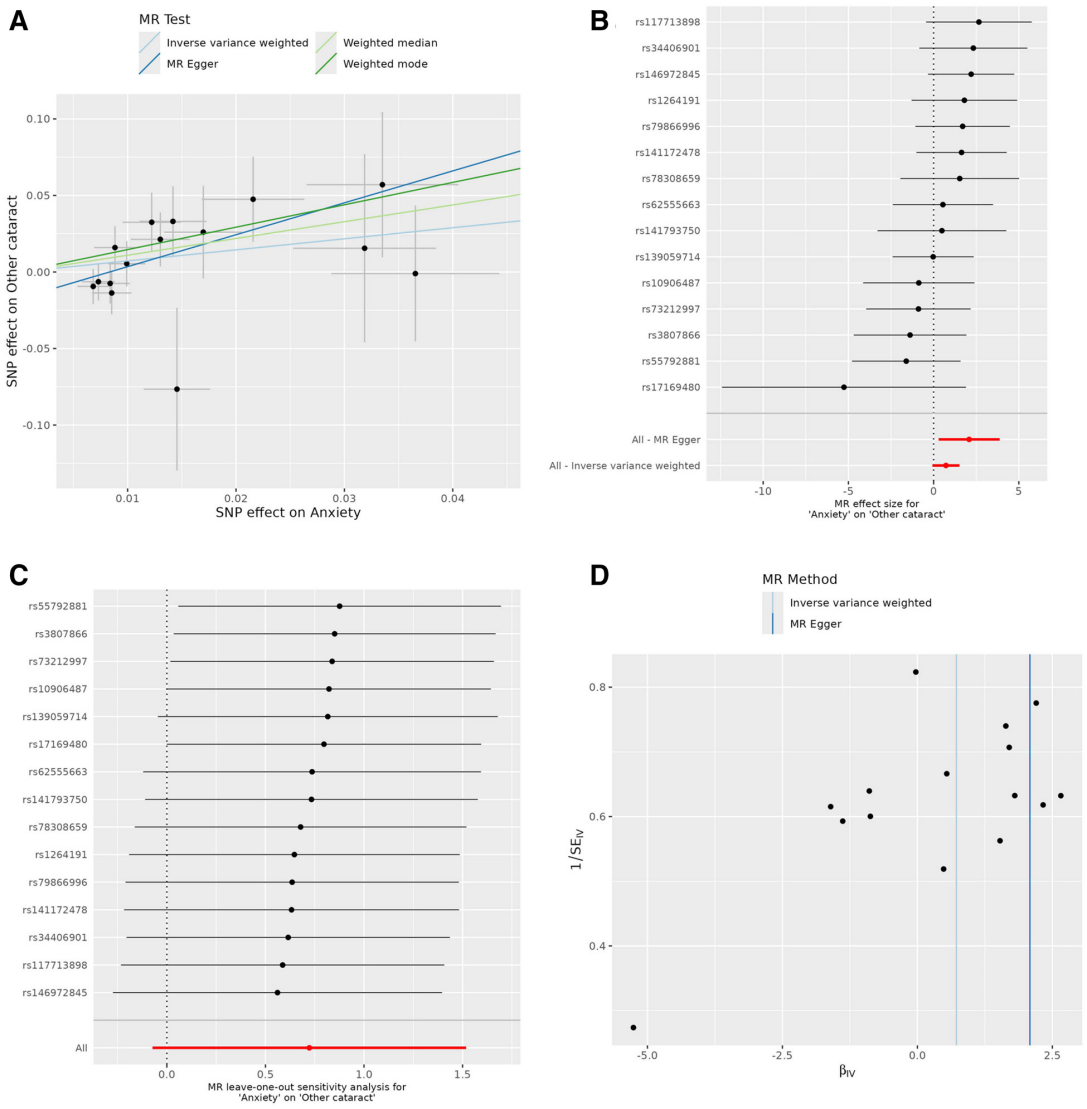
Causal effect of anxiety on other cataracts in the reverse MR analysis. (A) Scatter plot of the association between anxiety and other cataracts. IVW, MR-Egger, weighted median, and weighted mode methods were applied. (B) A forest plot was used to show the MR estimate and 95% CI values (gray line segment) for each SNP, which also shows the IVW and MR-Egger results at the bottom. (C) Leave-one-out analysis to evaluate whether any single instrumental variable was driving the causal effect of anxiety on other cataracts. (D) A funnel plot was applied to detect whether the observed association was accompanied by obvious heterogeneity. CI = confidence interval, IVW = inverse-variance weighted, MR = Mendelian randomization, SNPs = single-nucleotide polymorphisms.

In the reverse MR analysis, the IVW analysis did not reveal significant evidence of a causal relationship between psychiatric disorders and the risk associated with cataract (Table 1). This finding was further supported by additional methods, except for the relationship between other cataracts and anxiety disorder, which showed significant results in the MR-Egger (OR = 8.04, 95% CI: 1.34–48.3, *P* = .040) and weighted median (OR = 2.99, 95% CI: 1.02–8.79, *P* = .047) analyses, as detailed in Table 1 and Figure 3A and B. The relationship between other cataracts and anxiety disorder was further supported by leave-one-out sensitivity analysis (Fig. 3C), with the funnel plot (Fig. 3D; Fig. S2, Supplementary Digital Content 5) suggesting the association is unbiased. Cochran *Q* test indicated heterogeneity in the relationships between senile cataract and bipolar disorder (*Q* = 63.925, *P* = .003) and senile cataract and schizophrenia (*Q* = 82.585, *P* < .001, Table 2). The MR-Egger analysis suggested pleiotropy in the relationship between bipolar disorder and other cataracts (intercept = −0.032, *P* = .038, Table 2). The MR-PRESSO analysis indicated that the relationships between schizophrenia and senile cataract (global *P* < .001), schizophrenia and other cataracts (global *P* = .006), and bipolar disorder and other cataracts (global *P* = .019) were influenced by horizontal pleiotropy (Table 3). Of note, the MR-PRESSO method identified outliers for the associations between schizophrenia and senile cataract, schizophrenia and other cataracts (rs13195636), and bipolar disorder and other cataracts (rs10744560); however, after their removal, these relationships remained statistically insignificant (all *P* > .05, Table 3).

**Table 2:** Results of heterogeneity test and pleiotropy test of instrumental variables.

Exposure	Outcome	Hetergeneity		Pleiotropy	
		*Q* statistic (IVW)	*P*	MR-Egger intercept	*P*
Anxiety	Other cataract	14.097	.443	−0.017	.121
Other cataract	Anxiety	12.948	.531	0.001	.593
Anxiety	Senile cataract	14.987	.379	−0.004	.591
Senile cataract	Anxiety	48.205	.124	0.001	.53
Bipolar disorder	Other cataract	23.482	.015	−0.052	.136
Other cataract	Bipolar disorder	19.244	.156	−0.032	.038
Bipolar disorder	Senile cataract	15.027	.181	−0.018	.345
Senile cataract	Bipolar disorder	63.925	.003	−0.004	.646
Major depressive disorder	Other cataract	4.184	.242	−0.07	.211
Other cataract	Major depressive disorder	21.63	.087	−0.004	.726
Major depressive disorder	Senile cataract	4.414	.353	−0.041	.183
Senile cataract	Major depressive disorder	47.894	.089	−0.004	.423
Schizophrenia	Other cataract	193.851	.008	0	.917
Schizophrenia	Senile cataract	230.207	<.001	0	.979
Senile cataract	Schizophrenia	82.585	<.001	0.005	.531

IVW = inverse-variance weighted, MR = Mendelian randomization.

**Table 3:** The results of MR-PRESSO analysis.

Exposure	Outcome	Raw (uncorrected)		Outlier corrected		Global *P*	Number of outliers	Distortion *P*
		OR (95% CI)	*P*	OR (95% CI)	*P*			
Anxiety	Other cataracts	2.06 (0.93–4.56)	0.097	/	/	.462	/	/
Other cataract	Anxiety	0.99 (0.98–1)	0.031	/	/	.585	/	/
Anxiety	Senile cataract	1.56 (0.91–2.69)	0.131	/	/	.418	/	/
Senile cataract	Anxiety	1 (0.99–1.01)	0.831	/	/	.130	/	/
Biploar disorder	Other cataracts	0.99 (0.89–1.1)	0.805	1.02 (0.93–1.12)	.672	.019	1: rs10744560	.200
Other cataract	Biploar disorder	1 (0.9–1.11)	0.960	/	/	.162	/	/
Biploar disorder	Senile cataract	0.98 (0.92–1.04)	0.481	/	/	.146	/	/
Senile cataract	Biploar disorder	0.97 (0.88–1.08)	0.581	/	/	<.001	/	/
Major depressive disorder	Other cataracts	1.05 (0.76–1.44)	0.801	/	/	.095	/	/
Other cataract	Major depressive disorder	1.01 (0.94–1.07)	0.833	/	/	.134	/	/
Major depressive disorder	Senile cataract	1.06 (0.92–1.23)	0.459	/	/	.431	/	/
Senile cataract	Major depressive disorder	0.98 (0.93–1.04)	0.506	/	/	.045	/	/
Schizophrenia	Other cataracts	1.01 (0.98–1.05)	0.525	1 (0.97–1.04)	.801	.006	1: rs13195636	.308
Other cataract	Schizophrenia	0.97 (0.91–1.03)	0.285	/	/	.473	/	/
Schizophrenia	Senile cataract	1.01 (0.99–1.04)	0.371	1.01 (0.99–1.03)	.436	<.001	2	.839
Senile cataract	Schizophrenia	1.01 (0.93–1.09)	0.831	0.97 (0.91–1.04)	.416	<.001	2: rs12314392 & rs3094577	.327

CI = confidence interval, IVW = inverse-variance weighted, MR = Mendelian randomization, MR-PRESSO = MR pleiotropy residual sum and outlier, OR = odds ratio, Outlier corrected = MR-PRESSO estimates after removal of identified horizontal pleiotropic outliers; Raw (uncorrected) = MR-PRESSO estimates obtained without outlier correction.

## 4. Discussion

Our 2-sample MR study was designed to explore the causal relationship between cataract and psychiatric disorders. The MR analyses, employing the IVW method, demonstrated a significant causal association between other cataract and anxiety. Importantly, sensitivity analyses confirmed that this association is robust against heterogeneity and horizontal pleiotropy.

Cataract, defined by the opacity of the eye’s natural crystalline lens, is the foremost cause of blindness globally, contributing to nearly 42% of all blindness cases.^[28,29]^ It is influenced by a multitude of factors, including aging, eye injury, diabetes mellitus, ultraviolet exposure, medication use, and other ocular conditions.^[30–32]^ Among these, aging is the predominant cause, particularly in the form of senile cataract. Several studies have underscored the role of cataract as a risk factor for mental illnesses, revealing a significant correlation between cataract in children and adolescents and a heightened prevalence of psychiatric disorders.^[6–8,33]^ Mechanistically, cataracts and psychiatric disorders are interconnected through several biological pathways. For example, chronic psychological stress and psychiatric conditions increase oxidative stress, leading to the production of reactive oxygen species that may damage lens proteins and promote cataract formation.^[34]^ More specifically, oxidative stress activates genes encoding key antioxidants, such as glutathione S-transferases, superoxide dismutases, and peroxiredoxins.^[35]^ These protective mechanisms are upregulated by pathways mediated by transcription factors, including nuclear factor erythroid 2-related factor 2.^[36]^ However, these defense mechanisms could be overwhelmed and become dysfunctional, leading to oxidative damage to crystallins and other lens proteins.^[37]^ On the other hand, systemic low-grade inflammation, common in mental illnesses, also contributes to lens opacification via inflammatory mediators and cytokines, such as interleukin-6 and C-reactive protein.^[38]^ Our research, however, distinctively associates other cataracts with anxiety, positing that this category of cataract may attenuate the risk of developing this mental health issue. This is in contrast to studies highlighting the mental health burden experienced by cataract patients, particularly in children and adolescents, where cataract is associated with a higher incidence of psychiatric disorders.^[6,7]^ One plausible explanation is that cataract, being a common and treatable eye condition, often results in favorable outcomes due to established surgical interventions. This can alleviate anxiety as patients anticipate improved vision post-surgery.^[39,40]^ The high success rate of cataract surgery, which significantly enhances vision in most patients, may instill hope and mitigate the psychological impact of the disease. Additionally, cataract might alter visual perception, affecting how the brain processes emotional information, thereby indirectly influencing anxiety levels.^[41]^

It is noteworthy that MR-Egger and weighted median methods indicate a potential causal link between other cataract and bipolar disorder in the forward MR analysis. However, this association is not supported by the IVW method. This suggests that the potential role of other cataract in the development of bipolar disorder still warrants further exploration. Recent studies have begun to elucidate the relationship between cataract and bipolar disorder. For instance, Chu et al reported an increased risk of cataract development among patients with bipolar disorder and schizophrenia who have been on long-term treatment with lithium, lithium in combination with other mood stabilizers, and valproic acid in combination with other mood stabilizers for over 2 years.^[42]^ This suggests that the use of certain medications, particularly mood stabilizers, may be a contributing factor to the development of cataract in these patient populations. Furthermore, Sinha documented an unexpected case of cataract formation following treatment with valproic acid,^[43]^ adding to the body of evidence that implicates long-term medication use as a potential risk factor for cataract development in patients with bipolar disorder. Therefore, the observed association between other cataract and bipolar disorder may be attributed, at least in part, to the long-term use of medications commonly prescribed for mood disorders. However, this hypothesis requires further investigation through rigorous research to disentangle the complex interplay between medication use, disease progression, and the development of cataract.

Notably, our reverse MR analysis indicated a potential causal relationship between anxiety and other cataract by the MR-Egger and weighted median methods. Although the IVW method did not find a causal relationship between anxiety and other cataract, the causal relationship between them remains to be explored. The forward MR suggesting a protective effect of other cataract on anxiety is interpreted conservatively due to its lack of biological support. For the minimal protective effect observed in forward MR, the high success rate of cataract surgery and subsequent vision improvement may alleviate anxiety by instilling hope and reducing psychological distress. Alternatively, this counterintuitive result may be influenced by methodological limitations, including horizontal pleiotropy, residual confounding by lifestyle factors, or potential ascertainment bias related to increased medical surveillance in cataract patients. Conversely, in this reverse MR analysis, chronic stress associated with anxiety disorders may lead to physiological changes throughout the body, including the eyes. Specifically, the continuous release of stress hormones such as cortisol may potentially impact the eyes, contributing to opacification.^[44,45]^ Additionally, our research did not uncover a connection between senile cataract and mental illness. Senile cataract, an age-related condition experienced by nearly all elderly individuals, arises from a complex interplay of factors such as oxidative damage, nutrient deficiencies, and metabolic disorders, rather than psychological factors alone.^[46–48]^ Consequently, the relationship between senile cataract and mental illness may not be as pronounced as with other cataract types. Thus, while our results underscore the intricate interplay between cataract and psychiatric disorders, further research is warranted to elucidate these mechanisms, including the role of treatment outcomes and stress-induced physiological changes.

The use of MR in our study offers a significant advantage over traditional observational research by providing a causal estimate that is less susceptible to reverse causality and confounding bias. The extensive genome-wide association study data utilized in our MR analysis enhance its precision. However, our study is not without limitations. Firstly, the relaxed significance threshold for IV selection, while broadening the candidate pool, could introduce biases. Secondly, the static nature of genetic information in MR studies might overlook dynamic changes in protein expression influenced by age, gender, or other physiological factors, which could significantly impact disease risk. Thirdly, potential confounding variables, such as long-term medications, could lead to spurious associations, which could bias our causal estimates. Fourthly, limitations in capturing indirect effects via MR methodology are noteworthy. MR primarily assesses direct causal effects, and our study may not fully account for complex biological pathways involving multiple steps or feedback loops that could influence the relationship between the exposure and the outcome. Fifthly, possible biases introduced by sample overlap could lead to the overestimation of causal effects due to shared genetic variants. Moreover, the generalizability of our findings to other ethnic groups is uncertain, as our study population was predominantly European.

In conclusion, our MR study provides evidence supporting a causal relationship between non-senile cataract and anxiety, contributing to a deeper understanding of the underlying pathophysiological mechanisms and potentially informing targeted therapies. Clinically, while routine screening for major psychiatric disorders may not be warranted for patients with senile cataract, the link between non-senile cataract and anxiety highlights the importance for ophthalmologists to consider potential mental health comorbidities in patients with nonage-related cataracts. Future research should aim to replicate these findings in larger, diverse populations and explore the temporal relationship through longitudinal studies. Investigating the underlying biological pathways, such as inflammation and oxidative stress, through molecular studies would be crucial to understanding the underlying mechanisms behind these 2 conditions.

## Author contributions

**Conceptualization:** Xiaoling Su.

**Data curation:** Xiaoling Su.

**Formal analysis:** Xiaoling Su.

**Writing – original draft:** Xiaoling Su, Guoqiang Tian.

**Writing – review & editing:** Xiaoling Su, Guoqiang Tian.










