# Supplementary material for: Causal association between cataract and psychiatric disorders: A bidirectional 2-sample Mendelian randomization study
Source: Medicine (Baltimore). 2026 Aug 7;105(32):e50075. doi: 10.1097/MD.0000000000050075 (PMC13456826; doi:10.1097/MD.0000000000050075)
Supplement: Supplementary file 1 [file medi-105-e50075-s001.docx]

**Supplemental digital content table 1 Overview of the source of outcome and exposure data**

| **Trait** | **GWAS ID** | **Sample(case/control)** | **SNPs** |
| --- | --- | --- | --- |
| Major Depressive Disorder | ieu-a-1188 | 59,851/113,154 | 13,554,550 |
| Biopolar Disorder | ieu-b-41 | 20,352/31,358 | 13,413,244 |
| Schizophrenia | ieu-b-5102 | 52,017/75,889 | NA |
| Mental health problems ever diagnosed by a professional: Anxiety, nerves or generalized anxiety disorder | ukb-d-20544_15 | 117,751 | 13,571,547 |
| Senile cataract | H7_CATARACTSENILE | 65,235/341,546 | NA |
| Other cataract | H7_CATARACTOTHER | 19,401/341,546 | NA |
