# Supplementary material for: Causal association between cataract and psychiatric disorders: A bidirectional 2-sample Mendelian randomization study
Source: Medicine (Baltimore). 2026 Aug 7;105(32):e50075. doi: 10.1097/MD.0000000000050075 (PMC13456826; doi:10.1097/MD.0000000000050075)
Supplement: Supplementary file 2 [file medi-105-e50075-s002.docx]

**Supplementary digital content table 2** SNPs identified in the forward MR analysis

| chr.exposure | pos.exposure | effect_allele.exposure | other_allele.exposure | SNP | pval.exposure | beta.exposure | se.exposure | eaf.exposure | ncase.exposure | ncontrol.exposure | exposure | mr_keep.exposure | pval_origin.exposure | samplesize.exposure | id.exposure | r2 | F |
| --- | --- | --- | --- | --- | --- | --- | --- | --- | --- | --- | --- | --- | --- | --- | --- | --- | --- |
| 1 | 10762751 | T | C | rs2073017 | 1.28E-11 | -0.05617 | 0.008297 | 0.729899 | 0.724334 | 0.730962 | Senile cataract | TRUE | reported | 406781 | vuSBAC | 0.000113 | 45.84166 |
| 1 | 1.69E+08 | T | C | rs1200105 | 2.35E-09 | -0.04619 | 0.007735 | 0.651862 | 0.646474 | 0.652892 | Senile cataract | TRUE | reported | 406781 | vuSBAC | 8.77E-05 | 35.66315 |
| 1 | 2.27E+08 | A | T | rs2183836 | 6.01E-10 | 0.046566 | 0.007522 | 0.394857 | 0.401074 | 0.393669 | Senile cataract | TRUE | reported | 406781 | vuSBAC | 9.42E-05 | 38.3193 |
| 2 | 1.36E+08 | T | C | rs10193587 | 3.92E-08 | 0.046241 | 0.008416 | 0.261918 | 0.265416 | 0.26125 | Senile cataract | TRUE | reported | 406781 | vuSBAC | 7.42E-05 | 30.18967 |
| 2 | 1.43E+08 | T | C | rs1993689 | 1.75E-18 | -0.07998 | 0.009117 | 0.212522 | 0.203353 | 0.214274 | Senile cataract | TRUE | reported | 406781 | vuSBAC | 0.000189 | 76.95304 |
| 3 | 1.82E+08 | C | T | rs9842371 | 7.76E-58 | 0.129262 | 0.008063 | 0.288947 | 0.306051 | 0.285681 | Senile cataract | TRUE | reported | 406781 | vuSBAC | 0.000631 | 256.9912 |
| 5 | 1.33E+08 | G | A | rs147570901 | 1.21E-08 | -0.13099 | 0.02299 | 0.027769 | 0.026081 | 0.028091 | Senile cataract | TRUE | reported | 406781 | vuSBAC | 7.98E-05 | 32.46371 |
| 6 | 29566093 | A | G | rs3094577 | 1.75E-10 | 0.050445 | 0.007905 | 0.682163 | 0.686841 | 0.681269 | Senile cataract | TRUE | reported | 406781 | vuSBAC | 0.0001 | 40.72361 |
| 6 | 32636375 | A | G | rs9272324 | 6.98E-18 | 0.064273 | 0.00746 | 0.424178 | 0.4298 | 0.423104 | Senile cataract | TRUE | reported | 406781 | vuSBAC | 0.000182 | 74.2232 |
| 6 | 1.63E+08 | G | A | rs4709713 | 1.52E-13 | -0.05447 | 0.007375 | 0.498927 | 0.491526 | 0.50034 | Senile cataract | TRUE | reported | 406781 | vuSBAC | 0.000134 | 54.54502 |
| 7 | 11638151 | T | C | rs2526100 | 1.02E-11 | 0.069268 | 0.010181 | 0.151555 | 0.157235 | 0.15047 | Senile cataract | TRUE | reported | 406781 | vuSBAC | 0.000114 | 46.28699 |
| 7 | 28507989 | T | C | rs10258092 | 4.94E-12 | 0.0707 | 0.010236 | 0.151327 | 0.155259 | 0.150576 | Senile cataract | TRUE | reported | 406781 | vuSBAC | 0.000117 | 47.70875 |
| 7 | 46174220 | C | T | rs4559153 | 3.31E-13 | 0.06479 | 0.008898 | 0.218733 | 0.223617 | 0.2178 | Senile cataract | TRUE | reported | 406781 | vuSBAC | 0.00013 | 53.0158 |
| 7 | 1.27E+08 | G | A | rs62621812 | 3.31E-17 | 0.153825 | 0.018236 | 0.042321 | 0.045354 | 0.041741 | Senile cataract | TRUE | reported | 406781 | vuSBAC | 0.000175 | 71.14977 |
| 8 | 22672182 | A | G | rs4872531 | 3.53E-13 | 0.057554 | 0.007914 | 0.682572 | 0.68976 | 0.681199 | Senile cataract | TRUE | reported | 406781 | vuSBAC | 0.00013 | 52.89025 |
| 9 | 22206988 | C | T | rs1679013 | 9.89E-14 | 0.05562 | 0.007473 | 0.432767 | 0.439919 | 0.431401 | Senile cataract | TRUE | reported | 406781 | vuSBAC | 0.000136 | 55.38784 |
| 10 | 28735201 | G | A | rs1014607 | 1.97E-10 | -0.0635 | 0.009978 | 0.164786 | 0.160719 | 0.165563 | Senile cataract | TRUE | reported | 406781 | vuSBAC | 9.95E-05 | 40.49543 |
| 10 | 1E+08 | C | T | rs150832287 | 1.41E-09 | 0.15876 | 0.026222 | 0.019426 | 0.021325 | 0.019064 | Senile cataract | TRUE | reported | 406781 | vuSBAC | 9.01E-05 | 36.65516 |
| 10 | 1E+08 | G | C | rs2275657 | 1.45E-11 | -0.05004 | 0.00741 | 0.452924 | 0.446267 | 0.454195 | Senile cataract | TRUE | reported | 406781 | vuSBAC | 0.000112 | 45.59587 |
| 10 | 1.14E+08 | T | C | rs141266925 | 2.65E-15 | 0.240995 | 0.030481 | 0.014833 | 0.01586 | 0.014637 | Senile cataract | TRUE | reported | 406781 | vuSBAC | 0.000154 | 62.51086 |
| 10 | 1.22E+08 | C | T | rs60401382 | 8.55E-17 | 0.069358 | 0.008333 | 0.263516 | 0.271831 | 0.261927 | Senile cataract | TRUE | reported | 406781 | vuSBAC | 0.00017 | 69.27883 |
| 11 | 69179313 | C | T | rs57096576 | 8.38E-14 | 0.114041 | 0.015278 | 0.059904 | 0.064611 | 0.059004 | Senile cataract | TRUE | reported | 406781 | vuSBAC | 0.000137 | 55.71543 |
| 11 | 69491627 | T | A | rs654829 | 1.06E-08 | 0.043109 | 0.007536 | 0.604361 | 0.609773 | 0.603328 | Senile cataract | TRUE | reported | 406781 | vuSBAC | 8.04E-05 | 32.72259 |
| 12 | 30609549 | G | A | rs7135149 | 5.24E-13 | -0.05557 | 0.007698 | 0.3586 | 0.350715 | 0.360106 | Senile cataract | TRUE | reported | 406781 | vuSBAC | 0.000128 | 52.11237 |
| 12 | 1.1E+08 | A | G | rs12314392 | 1.68E-15 | 0.059515 | 0.007474 | 0.410032 | 0.417122 | 0.408678 | Senile cataract | TRUE | reported | 406781 | vuSBAC | 0.000156 | 63.41011 |
| 13 | 26464847 | A | G | rs4631994 | 5.29E-15 | 0.057863 | 0.007399 | 0.454008 | 0.462901 | 0.45231 | Senile cataract | TRUE | reported | 406781 | vuSBAC | 0.00015 | 61.15034 |
| 15 | 28111713 | C | T | rs1129038 | 6.05E-13 | -0.08362 | 0.011615 | 0.880952 | 0.876102 | 0.881878 | Senile cataract | TRUE | reported | 406781 | vuSBAC | 0.000127 | 51.82995 |
| 15 | 28702698 | A | C | rs80303640 | 2.29E-08 | 0.065411 | 0.011704 | 0.121834 | 0.124874 | 0.121253 | Senile cataract | TRUE | reported | 406781 | vuSBAC | 7.68E-05 | 31.23288 |
| 15 | 61506544 | G | A | rs4438253 | 1.05E-08 | 0.042352 | 0.0074 | 0.513221 | 0.520344 | 0.511861 | Senile cataract | TRUE | reported | 406781 | vuSBAC | 8.05E-05 | 32.75117 |
| 15 | 73931230 | T | C | rs28617339 | 1.50E-24 | -0.07527 | 0.00736 | 0.523738 | 0.514425 | 0.525517 | Senile cataract | TRUE | reported | 406781 | vuSBAC | 0.000257 | 104.5889 |
| 16 | 69521973 | A | T | rs889400 | 7.58E-11 | -0.05181 | 0.00796 | 0.687644 | 0.681412 | 0.688834 | Senile cataract | TRUE | reported | 406781 | vuSBAC | 0.000104 | 42.36324 |
| 16 | 80498797 | A | G | rs56055503 | 9.36E-10 | 0.050246 | 0.00821 | 0.282192 | 0.285309 | 0.281596 | Senile cataract | TRUE | reported | 406781 | vuSBAC | 9.21E-05 | 37.45398 |
| 17 | 9095448 | G | A | rs9902550 | 4.02E-09 | 0.046503 | 0.007904 | 0.679786 | 0.684779 | 0.678832 | Senile cataract | TRUE | reported | 406781 | vuSBAC | 8.51E-05 | 34.61302 |
| 17 | 31946703 | C | T | rs11657487 | 4.71E-09 | 0.04437 | 0.007576 | 0.387768 | 0.393107 | 0.386748 | Senile cataract | TRUE | reported | 406781 | vuSBAC | 8.43E-05 | 34.30384 |
| 17 | 43435239 | C | T | rs12950982 | 4.16E-09 | -0.04661 | 0.00793 | 0.318406 | 0.313352 | 0.319372 | Senile cataract | TRUE | reported | 406781 | vuSBAC | 8.49E-05 | 34.5477 |
| 17 | 76710298 | C | T | rs9909380 | 3.13E-09 | 0.044592 | 0.007526 | 0.395597 | 0.400396 | 0.394681 | Senile cataract | TRUE | reported | 406781 | vuSBAC | 8.63E-05 | 35.10265 |
| 17 | 81654604 | A | G | rs7405790 | 1.84E-10 | -0.04744 | 0.007443 | 0.573624 | 0.570043 | 0.574308 | Senile cataract | TRUE | reported | 406781 | vuSBAC | 9.99E-05 | 40.63101 |
| 18 | 55291010 | A | G | rs79052447 | 1.13E-10 | 0.121799 | 0.018889 | 0.039219 | 0.041999 | 0.038688 | Senile cataract | TRUE | reported | 406781 | vuSBAC | 0.000102 | 41.57748 |
| 19 | 44884873 | G | A | rs34342646 | 6.73E-11 | -0.06322 | 0.009686 | 0.182622 | 0.171763 | 0.184696 | Senile cataract | TRUE | reported | 406781 | vuSBAC | 0.000105 | 42.59696 |
| 20 | 10664549 | A | G | rs6040069 | 1.81E-09 | -0.05806 | 0.009654 | 0.82272 | 0.817081 | 0.823797 | Senile cataract | TRUE | reported | 406781 | vuSBAC | 8.89E-05 | 36.17284 |
| 20 | 19476624 | A | G | rs4814857 | 1.26E-41 | 0.142451 | 0.01054 | 0.138513 | 0.14929 | 0.136455 | Senile cataract | TRUE | reported | 406781 | vuSBAC | 0.000449 | 182.6791 |
| 21 | 17485236 | A | C | rs2849883 | 1.59E-08 | 0.042279 | 0.007481 | 0.406939 | 0.414335 | 0.405527 | Senile cataract | TRUE | reported | 406781 | vuSBAC | 7.85E-05 | 31.93836 |
| 2 | 1.43E+08 | G | A | rs34794422 | 2.84E-13 | -0.1003 | 0.013736 | 0.213834 | 0.201575 | 0.21453 | Other cataract | TRUE | reported | 360947 | uSG04e | 0.000148 | 53.31825 |
| 3 | 1.82E+08 | G | A | rs6414493 | 1.21E-12 | 0.086688 | 0.012202 | 0.285791 | 0.297446 | 0.285128 | Other cataract | TRUE | reported | 360947 | uSG04e | 0.00014 | 50.47046 |
| 6 | 31496252 | C | T | rs2534676 | 1.85E-13 | 0.083456 | 0.01134 | 0.604597 | 0.618647 | 0.603799 | Other cataract | TRUE | reported | 360947 | uSG04e | 0.00015 | 54.15947 |
| 6 | 32372293 | C | T | rs1980496 | 5.48E-23 | 0.119201 | 0.012074 | 0.295955 | 0.312375 | 0.295023 | Other cataract | TRUE | reported | 360947 | uSG04e | 0.00027 | 97.46643 |
| 6 | 32834685 | G | A | rs62397688 | 4.08E-09 | 0.114232 | 0.019424 | 0.08725 | 0.092805 | 0.086935 | Other cataract | TRUE | reported | 360947 | uSG04e | 9.58E-05 | 34.58564 |
| 7 | 28498843 | G | T | rs60030925 | 2.25E-08 | 0.085732 | 0.015332 | 0.150961 | 0.158487 | 0.150533 | Other cataract | TRUE | reported | 360947 | uSG04e | 8.66E-05 | 31.2665 |
| 7 | 46174220 | C | T | rs4559153 | 4.54E-12 | 0.091875 | 0.013278 | 0.218491 | 0.23066 | 0.2178 | Other cataract | TRUE | reported | 360947 | uSG04e | 0.000133 | 47.87742 |
| 7 | 1.27E+08 | G | A | rs62621812 | 2.87E-13 | 0.194671 | 0.026665 | 0.042131 | 0.049002 | 0.041741 | Other cataract | TRUE | reported | 360947 | uSG04e | 0.000148 | 53.29754 |
| 10 | 1E+08 | G | A | rs1502593 | 3.18E-08 | -0.06161 | 0.011138 | 0.471378 | 0.459936 | 0.472028 | Other cataract | TRUE | reported | 360947 | uSG04e | 8.48E-05 | 30.5944 |
| 10 | 1.02E+08 | G | A | rs2273555 | 1.15E-09 | -0.06886 | 0.011313 | 0.608977 | 0.595727 | 0.60973 | Other cataract | TRUE | reported | 360947 | uSG04e | 0.000103 | 37.04742 |
| 10 | 1.14E+08 | T | C | rs141266925 | 9.46E-18 | 0.370223 | 0.043148 | 0.014871 | 0.018983 | 0.014637 | Other cataract | TRUE | reported | 360947 | uSG04e | 0.000204 | 73.62157 |
| 10 | 1.22E+08 | C | T | rs11200643 | 1.11E-16 | 0.103581 | 0.012491 | 0.261238 | 0.277851 | 0.260294 | Other cataract | TRUE | reported | 360947 | uSG04e | 0.00019 | 68.76215 |
| 15 | 27983407 | C | T | rs74653330 | 5.08E-12 | 0.180583 | 0.026159 | 0.043708 | 0.050096 | 0.043345 | Other cataract | TRUE | reported | 360947 | uSG04e | 0.000132 | 47.65433 |
| 15 | 28250810 | A | G | rs12912427 | 3.90E-09 | -0.10848 | 0.018423 | 0.896731 | 0.889007 | 0.897169 | Other cataract | TRUE | reported | 360947 | uSG04e | 9.61E-05 | 34.67216 |
| 17 | 81719522 | G | C | rs11868178 | 1.50E-10 | -0.07329 | 0.011443 | 0.566644 | 0.553347 | 0.567399 | Other cataract | TRUE | reported | 360947 | uSG04e | 0.000114 | 41.0273 |
| 18 | 55783633 | C | T | rs72932578 | 1.26E-08 | 0.177634 | 0.031214 | 0.02985 | 0.034844 | 0.029566 | Other cataract | TRUE | reported | 360947 | uSG04e | 8.97E-05 | 32.38552 |
| 20 | 19476624 | A | G | rs4814857 | 1.38E-21 | 0.150036 | 0.015721 | 0.137312 | 0.152395 | 0.136455 | Other cataract | TRUE | reported | 360947 | uSG04e | 0.000252 | 91.08225 |
