# Supplementary material for: Causal association between cataract and psychiatric disorders: A bidirectional 2-sample Mendelian randomization study
Source: Medicine (Baltimore). 2026 Aug 7;105(32):e50075. doi: 10.1097/MD.0000000000050075 (PMC13456826; doi:10.1097/MD.0000000000050075)
Supplement: Supplementary file 3 [file medi-105-e50075-s003.docx]

**Supplementary digital content table 3** SNPs identified in the reverse MR analysis

| chr.exposure | samplesize.exposure | pval.exposure | pos.exposure | se.exposure | beta.exposure | id.exposure | SNP | effect_allele.exposure | other_allele.exposure | eaf.exposure | exposure | mr_keep.exposure | pval_origin.exposure | data_source.exposure | r2 | F |
| --- | --- | --- | --- | --- | --- | --- | --- | --- | --- | --- | --- | --- | --- | --- | --- | --- |
| 1 | 173005 | 2.15E-08 | 72837239 | 0.008 | 0.044997 | ieu-a-1188 | rs7531118 | C | T | 0.5537 | Major Depressive Disorder | TRUE | reported | igd | 0.000183 | 31.6366 |
| 1 | 173005 | 5.45E-09 | 73768366 | 0.0082 | 0.047799 | ieu-a-1188 | rs12129573 | A | C | 0.3966 | Major Depressive Disorder | TRUE | reported | igd | 0.000196 | 33.97884 |
| 5 | 173005 | 2.32E-11 | 103783801 | 0.0085 | 0.056702 | ieu-a-1188 | rs76025409 | C | G | 0.3579 | Major Depressive Disorder | TRUE | reported | igd | 0.000257 | 44.49874 |
| 6 | 173005 | 3.47E-11 | 28262686 | 0.0112 | -0.074 | ieu-a-1188 | rs6905391 | A | G | 0.1193 | Major Depressive Disorder | TRUE | reported | igd | 0.000252 | 43.65596 |
| 14 | 173005 | 8.15E-09 | 42097937 | 0.0079 | -0.0454 | ieu-a-1188 | rs1950829 | G | A | 0.5189 | Major Depressive Disorder | TRUE | reported | igd | 0.000191 | 33.03083 |
| 2 | 51710 | 5.92E-09 | 97413488 | 0.0144 | -0.0841 | ieu-b-41 | rs2314398 | G | C | 0.319554 | Biopolar Disorder | TRUE | reported | igd | 0.000659 | 34.10915 |
| 3 | 51710 | 5.53E-14 | 36856030 | 0.0134 | 0.101003 | ieu-b-41 | rs9834970 | C | T | 0.501233 | Biopolar Disorder | TRUE | reported | igd | 0.001098 | 56.81227 |
| 3 | 51710 | 9.09E-09 | 52847601 | 0.0135 | -0.0777 | ieu-b-41 | rs2071044 | T | C | 0.467916 | Biopolar Disorder | TRUE | reported | igd | 0.00064 | 33.12684 |
| 4 | 51710 | 3.27E-08 | 162294038 | 0.0188 | -0.1041 | ieu-b-41 | rs11724116 | T | C | 0.155277 | Biopolar Disorder | TRUE | reported | igd | 0.000593 | 30.65683 |
| 5 | 51710 | 1.54E-08 | 133906609 | 0.0139 | -0.0788 | ieu-b-41 | rs329319 | G | A | 0.565703 | Biopolar Disorder | TRUE | reported | igd | 0.000621 | 32.13868 |
| 6 | 51710 | 4.92E-08 | 50816718 | 0.0215 | 0.117096 | ieu-b-41 | rs55648125 | G | A | 0.108936 | Biopolar Disorder | TRUE | reported | igd | 0.000573 | 29.66132 |
| 6 | 51710 | 4.60E-08 | 166995260 | 0.0137 | 0.075004 | ieu-b-41 | rs10455979 | G | C | 0.473478 | Biopolar Disorder | TRUE | reported | igd | 0.000579 | 29.97167 |
| 7 | 51710 | 2.70E-08 | 24771777 | 0.0204 | 0.113202 | ieu-b-41 | rs17150022 | C | T | 0.120723 | Biopolar Disorder | TRUE | reported | igd | 0.000595 | 30.79152 |
| 7 | 51710 | 3.36E-08 | 110197412 | 0.0219 | -0.1207 | ieu-b-41 | rs13231398 | C | G | 0.107064 | Biopolar Disorder | TRUE | reported | igd | 0.000587 | 30.37453 |
| 11 | 51710 | 1.05E-08 | 79156748 | 0.019 | 0.108702 | ieu-b-41 | rs73496688 | A | T | 0.149117 | Biopolar Disorder | TRUE | reported | igd | 0.000633 | 32.73038 |
| 11 | 51710 | 3.66E-08 | 61618608 | 0.0141 | 0.0774 | ieu-b-41 | rs174592 | G | A | 0.372297 | Biopolar Disorder | TRUE | reported | igd | 0.000582 | 30.13193 |
| 12 | 51710 | 2.92E-09 | 2387099 | 0.014 | 0.083201 | ieu-b-41 | rs10744560 | T | C | 0.342297 | Biopolar Disorder | TRUE | reported | igd | 0.000683 | 35.31703 |
| 15 | 51710 | 1.93E-08 | 85153804 | 0.0146 | -0.0821 | ieu-b-41 | rs71395455 | G | A | 0.31149 | Biopolar Disorder | TRUE | reported | igd | 0.000611 | 31.61706 |
| 17 | 51710 | 5.80E-09 | 53367464 | 0.0138 | 0.080298 | ieu-b-41 | rs884301 | T | C | 0.381084 | Biopolar Disorder | TRUE | reported | igd | 0.000654 | 33.85591 |
| 19 | 51710 | 2.40E-10 | 19358207 | 0.0184 | 0.1166 | ieu-b-41 | rs111444407 | T | C | 0.154691 | Biopolar Disorder | TRUE | reported | igd | 0.000776 | 40.15546 |
| 22 | 51710 | 3.23E-08 | 41161049 | 0.0135 | -0.0744 | ieu-b-41 | rs5758065 | G | C | 0.489522 | Biopolar Disorder | TRUE | reported | igd | 0.000587 | 30.3728 |
| 1 | 130644 | 1.53E-12 | 239198959 | 0.0147 | 0.103895 | ieu-b-5102 | rs11587347 | G | C | 0.094 | Schizophrenia | TRUE | reported | igd | 0.000382 | 49.95144 |
| 1 | 130644 | 6.02E-12 | 30427639 | 0.0094 | -0.0647 | ieu-b-5102 | rs56335113 | G | A | 0.697 | Schizophrenia | TRUE | reported | igd | 0.000363 | 47.37614 |
| 1 | 130644 | 2.28E-18 | 73768366 | 0.0089 | 0.077799 | ieu-b-5102 | rs12129573 | A | C | 0.366 | Schizophrenia | TRUE | reported | igd | 0.000585 | 76.41275 |
| 1 | 130644 | 1.62E-12 | 243793012 | 0.012 | 0.085101 | ieu-b-5102 | rs145071536 | C | T | 0.192 | Schizophrenia | TRUE | reported | igd | 0.000385 | 50.29156 |
| 1 | 130644 | 3.08E-08 | 36627542 | 0.0092 | 0.051104 | ieu-b-5102 | rs4653164 | T | C | 0.668 | Schizophrenia | TRUE | reported | igd | 0.000236 | 30.8549 |
| 1 | 130644 | 3.56E-08 | 66331478 | 0.0088 | 0.048403 | ieu-b-5102 | rs1892346 | A | T | 0.564 | Schizophrenia | TRUE | reported | igd | 0.000232 | 30.25291 |
| 1 | 130644 | 1.73E-21 | 98552832 | 0.0108 | 0.102598 | ieu-b-5102 | rs1198588 | T | A | 0.788 | Schizophrenia | TRUE | reported | igd | 0.00069 | 90.2451 |
| 1 | 130644 | 7.99E-09 | 150115398 | 0.0116 | 0.066995 | ieu-b-5102 | rs12138231 | A | T | 0.817 | Schizophrenia | TRUE | reported | igd | 0.000255 | 33.35499 |
| 1 | 130644 | 1.84E-09 | 200414959 | 0.0089 | -0.0535 | ieu-b-5102 | rs7515363 | T | C | 0.619 | Schizophrenia | TRUE | reported | igd | 0.000277 | 36.13832 |
| 1 | 130644 | 7.19E-12 | 2373168 | 0.0091 | 0.062301 | ieu-b-5102 | rs6673880 | G | A | 0.495 | Schizophrenia | TRUE | reported | igd | 0.000359 | 46.87061 |
| 1 | 130644 | 5.75E-09 | 8431607 | 0.0087 | -0.051 | ieu-b-5102 | rs3795310 | T | C | 0.463 | Schizophrenia | TRUE | reported | igd | 0.000263 | 34.35955 |
| 1 | 130644 | 2.68E-12 | 44100084 | 0.0091 | -0.0635 | ieu-b-5102 | rs11210892 | A | G | 0.67 | Schizophrenia | TRUE | reported | igd | 0.000373 | 48.69281 |
| 1 | 130644 | 1.30E-10 | 97878068 | 0.0116 | 0.074303 | ieu-b-5102 | rs11165867 | T | C | 0.16 | Schizophrenia | TRUE | reported | igd | 0.000314 | 41.02936 |
| 1 | 130644 | 4.15E-12 | 177276006 | 0.0107 | 0.074497 | ieu-b-5102 | rs16851048 | C | T | 0.195 | Schizophrenia | TRUE | reported | igd | 0.000371 | 48.47378 |
| 2 | 130644 | 4.47E-16 | 199990107 | 0.0087 | 0.070895 | ieu-b-5102 | rs1451488 | G | A | 0.555 | Schizophrenia | TRUE | reported | igd | 0.000508 | 66.40219 |
| 2 | 130644 | 7.30E-14 | 200045258 | 0.0087 | -0.0649 | ieu-b-5102 | rs2167378 | T | C | 0.439 | Schizophrenia | TRUE | reported | igd | 0.000426 | 55.64355 |
| 2 | 130644 | 3.02E-08 | 212290048 | 0.0108 | -0.06 | ieu-b-5102 | rs3791710 | C | T | 0.203 | Schizophrenia | TRUE | reported | igd | 0.000236 | 30.86712 |
| 2 | 130644 | 2.49E-08 | 2327295 | 0.0097 | 0.054097 | ieu-b-5102 | rs6715366 | A | G | 0.267 | Schizophrenia | TRUE | reported | igd | 0.000238 | 31.1028 |
| 2 | 130644 | 1.47E-08 | 79426855 | 0.0091 | -0.0517 | ieu-b-5102 | rs6721531 | T | A | 0.341 | Schizophrenia | TRUE | reported | igd | 0.000247 | 32.27714 |
| 2 | 130644 | 8.28E-12 | 145183851 | 0.0129 | -0.0883 | ieu-b-5102 | rs13016542 | C | T | 0.136 | Schizophrenia | TRUE | reported | igd | 0.000359 | 46.85692 |
| 2 | 130644 | 2.36E-09 | 201253769 | 0.0095 | -0.057 | ieu-b-5102 | rs3739118 | A | G | 0.291 | Schizophrenia | TRUE | reported | igd | 0.000276 | 36.0045 |
| 2 | 130644 | 5.14E-11 | 22749726 | 0.0087 | -0.0574 | ieu-b-5102 | rs12712510 | C | T | 0.527 | Schizophrenia | TRUE | reported | igd | 0.000333 | 43.5299 |
| 2 | 130644 | 2.07E-08 | 97746526 | 0.0172 | -0.0963 | ieu-b-5102 | rs7575796 | G | A | 0.094 | Schizophrenia | TRUE | reported | igd | 0.00024 | 31.34689 |
| 2 | 130644 | 3.39E-08 | 156835793 | 0.0092 | -0.0507 | ieu-b-5102 | rs1881046 | T | G | 0.34 | Schizophrenia | TRUE | reported | igd | 0.000232 | 30.37233 |
| 2 | 130644 | 2.66E-09 | 172956449 | 0.0111 | -0.0661 | ieu-b-5102 | rs62183855 | C | A | 0.192 | Schizophrenia | TRUE | reported | igd | 0.000271 | 35.45741 |
| 2 | 130644 | 4.29E-10 | 185601420 | 0.0087 | -0.0544 | ieu-b-5102 | rs11693094 | T | C | 0.461 | Schizophrenia | TRUE | reported | igd | 0.000299 | 39.10214 |
| 2 | 130644 | 1.31E-12 | 198274929 | 0.0086 | -0.0611 | ieu-b-5102 | rs12151767 | A | G | 0.487 | Schizophrenia | TRUE | reported | igd | 0.000386 | 50.48273 |
| 2 | 130644 | 1.49E-17 | 233743109 | 0.0095 | 0.080603 | ieu-b-5102 | rs778371 | G | A | 0.286 | Schizophrenia | TRUE | reported | igd | 0.000551 | 71.98591 |
| 2 | 130644 | 4.28E-16 | 57961602 | 0.0087 | 0.070401 | ieu-b-5102 | rs13011472 | G | C | 0.484 | Schizophrenia | TRUE | reported | igd | 0.000501 | 65.48058 |
| 2 | 130644 | 5.35E-09 | 37575381 | 0.0091 | -0.0529 | ieu-b-5102 | rs3770754 | G | C | 0.368 | Schizophrenia | TRUE | reported | igd | 0.000259 | 33.78751 |
| 2 | 130644 | 2.74E-09 | 73837955 | 0.0102 | 0.060398 | ieu-b-5102 | rs6546857 | G | A | 0.232 | Schizophrenia | TRUE | reported | igd | 0.000268 | 35.06188 |
| 2 | 130644 | 1.48E-08 | 162845855 | 0.0087 | -0.049 | ieu-b-5102 | rs2909457 | A | G | 0.564 | Schizophrenia | TRUE | reported | igd | 0.000243 | 31.72062 |
| 3 | 130644 | 7.47E-11 | 16875823 | 0.0089 | 0.057905 | ieu-b-5102 | rs12489270 | C | T | 0.375 | Schizophrenia | TRUE | reported | igd | 0.000324 | 42.32914 |
| 3 | 130644 | 4.57E-11 | 63903759 | 0.0093 | 0.061099 | ieu-b-5102 | rs6798742 | G | A | 0.307 | Schizophrenia | TRUE | reported | igd | 0.00033 | 43.16155 |
| 3 | 130644 | 2.34E-08 | 107379837 | 0.009 | 0.050199 | ieu-b-5102 | rs167924 | G | A | 0.631 | Schizophrenia | TRUE | reported | igd | 0.000238 | 31.11014 |
| 3 | 130644 | 6.15E-10 | 17868759 | 0.0086 | 0.053295 | ieu-b-5102 | rs1430894 | T | C | 0.483 | Schizophrenia | TRUE | reported | igd | 0.000294 | 38.40381 |
| 3 | 130644 | 4.31E-08 | 30044778 | 0.0088 | -0.0483 | ieu-b-5102 | rs6549963 | C | T | 0.416 | Schizophrenia | TRUE | reported | igd | 0.000231 | 30.13003 |
| 3 | 130644 | 1.23E-19 | 52815905 | 0.0086 | -0.0784 | ieu-b-5102 | rs2710323 | C | T | 0.49 | Schizophrenia | TRUE | reported | igd | 0.000636 | 83.1146 |
| 3 | 130644 | 1.80E-11 | 2547786 | 0.0116 | 0.078199 | ieu-b-5102 | rs17194490 | T | G | 0.161 | Schizophrenia | TRUE | reported | igd | 0.000348 | 45.4448 |
| 3 | 130644 | 1.53E-08 | 71563777 | 0.0088 | -0.0496 | ieu-b-5102 | rs60135207 | T | G | 0.415 | Schizophrenia | TRUE | reported | igd | 0.000243 | 31.76734 |
| 3 | 130644 | 5.46E-11 | 136398387 | 0.0088 | 0.057703 | ieu-b-5102 | rs7634476 | G | A | 0.586 | Schizophrenia | TRUE | reported | igd | 0.000329 | 42.99612 |
| 3 | 130644 | 9.19E-12 | 36848316 | 0.0092 | 0.062503 | ieu-b-5102 | rs9876421 | T | C | 0.343 | Schizophrenia | TRUE | reported | igd | 0.000353 | 46.15551 |
| 3 | 130644 | 3.24E-08 | 117772036 | 0.014 | 0.077205 | ieu-b-5102 | rs1604060 | G | A | 0.885 | Schizophrenia | TRUE | reported | igd | 0.000233 | 30.4109 |
| 3 | 130644 | 8.83E-09 | 161487491 | 0.0087 | -0.0501 | ieu-b-5102 | rs308697 | A | C | 0.436 | Schizophrenia | TRUE | reported | igd | 0.000254 | 33.16597 |
| 3 | 130644 | 1.07E-12 | 180733150 | 0.0109 | -0.0775 | ieu-b-5102 | rs7647398 | T | C | 0.2 | Schizophrenia | TRUE | reported | igd | 0.000387 | 50.54989 |
| 4 | 130644 | 1.37E-08 | 706700 | 0.0089 | 0.050704 | ieu-b-5102 | rs35734242 | C | T | 0.426 | Schizophrenia | TRUE | reported | igd | 0.000248 | 32.45621 |
| 4 | 130644 | 2.69E-10 | 23423586 | 0.0091 | 0.057703 | ieu-b-5102 | rs215412 | A | G | 0.328 | Schizophrenia | TRUE | reported | igd | 0.000308 | 40.20794 |
| 4 | 130644 | 3.07E-08 | 31202669 | 0.0093 | -0.0516 | ieu-b-5102 | rs11941714 | A | G | 0.336 | Schizophrenia | TRUE | reported | igd | 0.000236 | 30.779 |
| 4 | 130644 | 4.10E-08 | 167974142 | 0.0088 | -0.0483 | ieu-b-5102 | rs1427633 | C | G | 0.593 | Schizophrenia | TRUE | reported | igd | 0.000231 | 30.13003 |
| 4 | 129529 | 2.90E-21 | 103188709 | 0.0168 | 0.158703 | ieu-b-5102 | rs13107325 | T | C | 0.067 | Schizophrenia | TRUE | reported | igd | 0.000688 | 89.23701 |
| 4 | 130644 | 1.25E-11 | 176859992 | 0.0105 | -0.0712 | ieu-b-5102 | rs2333321 | G | A | 0.792 | Schizophrenia | TRUE | reported | igd | 0.000352 | 45.98552 |
| 5 | 130644 | 4.38E-13 | 45252500 | 0.0092 | 0.066802 | ieu-b-5102 | rs10035564 | G | A | 0.331 | Schizophrenia | TRUE | reported | igd | 0.000403 | 52.72321 |
| 5 | 130644 | 2.68E-10 | 88743219 | 0.0104 | -0.0657 | ieu-b-5102 | rs16867571 | G | A | 0.235 | Schizophrenia | TRUE | reported | igd | 0.000305 | 39.91202 |
| 5 | 130644 | 4.66E-10 | 137892170 | 0.0088 | -0.055 | ieu-b-5102 | rs10117 | A | G | 0.398 | Schizophrenia | TRUE | reported | igd | 0.000299 | 39.06105 |
| 5 | 130644 | 5.72E-10 | 101723875 | 0.0094 | -0.0584 | ieu-b-5102 | rs1901512 | C | T | 0.699 | Schizophrenia | TRUE | reported | igd | 0.000295 | 38.59919 |
| 5 | 130644 | 2.03E-08 | 106766340 | 0.0119 | -0.0667 | ieu-b-5102 | rs187557 | T | C | 0.848 | Schizophrenia | TRUE | reported | igd | 0.00024 | 31.41187 |
| 5 | 130644 | 4.55E-13 | 152235215 | 0.0096 | -0.0692 | ieu-b-5102 | rs72802868 | T | G | 0.294 | Schizophrenia | TRUE | reported | igd | 0.000398 | 51.95852 |
| 5 | 130644 | 1.13E-09 | 153680747 | 0.0088 | 0.053696 | ieu-b-5102 | rs11740474 | T | A | 0.415 | Schizophrenia | TRUE | reported | igd | 0.000285 | 37.23189 |
| 5 | 130644 | 7.33E-09 | 139065988 | 0.0091 | 0.052599 | ieu-b-5102 | rs9687282 | G | T | 0.334 | Schizophrenia | TRUE | reported | igd | 0.000256 | 33.40967 |
| 5 | 130644 | 1.52E-08 | 155775075 | 0.0086 | -0.0488 | ieu-b-5102 | rs12652777 | C | T | 0.522 | Schizophrenia | TRUE | reported | igd | 0.000246 | 32.19814 |
| 5 | 130644 | 5.37E-16 | 60621839 | 0.0087 | 0.070197 | ieu-b-5102 | rs4700418 | G | C | 0.493 | Schizophrenia | TRUE | reported | igd | 0.000498 | 65.10201 |
| 6 | 130644 | 3.35E-08 | 70003389 | 0.0098 | -0.0544 | ieu-b-5102 | rs9454727 | G | A | 0.273 | Schizophrenia | TRUE | reported | igd | 0.000236 | 30.81675 |
| 6 | 130644 | 4.39E-11 | 73155285 | 0.0091 | -0.06 | ieu-b-5102 | rs2815731 | A | C | 0.358 | Schizophrenia | TRUE | reported | igd | 0.000333 | 43.47713 |
| 6 | 130644 | 1.54E-22 | 26090270 | 0.0173 | -0.1687 | ieu-b-5102 | rs34555420 | T | G | 0.075 | Schizophrenia | TRUE | reported | igd | 0.000727 | 95.08472 |
| 6 | 130644 | 1.78E-11 | 93077500 | 0.0099 | 0.066396 | ieu-b-5102 | rs634940 | T | G | 0.252 | Schizophrenia | TRUE | reported | igd | 0.000344 | 44.97896 |
| 6 | 130644 | 1.64E-09 | 33796794 | 0.0088 | 0.053295 | ieu-b-5102 | rs9461916 | C | T | 0.594 | Schizophrenia | TRUE | reported | igd | 0.000281 | 36.67802 |
| 6 | 130644 | 8.05E-09 | 84341803 | 0.0087 | -0.0503 | ieu-b-5102 | rs217336 | A | C | 0.434 | Schizophrenia | TRUE | reported | igd | 0.000256 | 33.43088 |
| 6 | 130644 | 6.55E-40 | 27509493 | 0.0159 | -0.2105 | ieu-b-5102 | rs13195636 | C | A | 0.091 | Schizophrenia | TRUE | reported | igd | 0.00134 | 175.2749 |
| 6 | 130644 | 8.47E-09 | 29748690 | 0.0096 | -0.0551 | ieu-b-5102 | rs1611236 | A | G | 0.314 | Schizophrenia | TRUE | reported | igd | 0.000252 | 32.9465 |
| 7 | 130644 | 3.49E-08 | 133128127 | 0.0134 | -0.074 | ieu-b-5102 | rs7798283 | G | T | 0.133 | Schizophrenia | TRUE | reported | igd | 0.000233 | 30.49878 |
| 7 | 130644 | 8.85E-14 | 137072531 | 0.009 | -0.0674 | ieu-b-5102 | rs728055 | A | T | 0.358 | Schizophrenia | TRUE | reported | igd | 0.000429 | 56.07744 |
| 7 | 130644 | 1.40E-08 | 71770973 | 0.0087 | -0.0491 | ieu-b-5102 | rs1914399 | G | C | 0.521 | Schizophrenia | TRUE | reported | igd | 0.000244 | 31.85632 |
| 7 | 130644 | 1.75E-08 | 87244960 | 0.0086 | -0.0487 | ieu-b-5102 | rs13233308 | T | C | 0.484 | Schizophrenia | TRUE | reported | igd | 0.000245 | 32.0725 |
| 7 | 130644 | 2.24E-24 | 2029867 | 0.0088 | -0.0896 | ieu-b-5102 | rs58120505 | C | T | 0.418 | Schizophrenia | TRUE | reported | igd | 0.000793 | 103.6748 |
| 7 | 130644 | 1.57E-15 | 86403263 | 0.0132 | -0.1051 | ieu-b-5102 | rs6943762 | C | T | 0.13 | Schizophrenia | TRUE | reported | igd | 0.000485 | 63.39199 |
| 7 | 130644 | 6.80E-10 | 110056000 | 0.0089 | -0.0549 | ieu-b-5102 | rs6974218 | C | A | 0.382 | Schizophrenia | TRUE | reported | igd | 0.000291 | 38.04378 |
| 7 | 130644 | 2.15E-11 | 110988593 | 0.0093 | -0.0623 | ieu-b-5102 | rs35426637 | T | G | 0.324 | Schizophrenia | TRUE | reported | igd | 0.000343 | 44.87274 |
| 7 | 130644 | 4.14E-10 | 24717969 | 0.0137 | 0.085602 | ieu-b-5102 | rs79210963 | C | T | 0.108 | Schizophrenia | TRUE | reported | igd | 0.000299 | 39.04046 |
| 7 | 130644 | 6.19E-15 | 104594253 | 0.0088 | 0.068504 | ieu-b-5102 | rs2252074 | G | T | 0.395 | Schizophrenia | TRUE | reported | igd | 0.000464 | 60.59769 |
| 7 | 130644 | 7.45E-09 | 131619847 | 0.0111 | 0.064101 | ieu-b-5102 | rs1593304 | G | A | 0.801 | Schizophrenia | TRUE | reported | igd | 0.000255 | 33.34887 |
| 8 | 130644 | 4.34E-13 | 27442127 | 0.0142 | -0.1026 | ieu-b-5102 | rs73229090 | A | C | 0.112 | Schizophrenia | TRUE | reported | igd | 0.000399 | 52.20695 |
| 8 | 130644 | 3.05E-09 | 144870701 | 0.0091 | -0.0538 | ieu-b-5102 | rs11136325 | A | G | 0.579 | Schizophrenia | TRUE | reported | igd | 0.000267 | 34.94796 |
| 8 | 130644 | 2.80E-08 | 33863561 | 0.0222 | 0.1234 | ieu-b-5102 | rs79445414 | C | T | 0.04 | Schizophrenia | TRUE | reported | igd | 0.000236 | 30.8971 |
| 8 | 130644 | 3.85E-10 | 60700469 | 0.0087 | -0.0547 | ieu-b-5102 | rs6984242 | A | G | 0.6 | Schizophrenia | TRUE | reported | igd | 0.000302 | 39.52519 |
| 8 | 130644 | 6.57E-09 | 89283689 | 0.0098 | -0.0571 | ieu-b-5102 | rs1915019 | G | A | 0.75 | Schizophrenia | TRUE | reported | igd | 0.00026 | 33.94604 |
| 8 | 130644 | 5.11E-18 | 143312933 | 0.0087 | -0.075 | ieu-b-5102 | rs4129585 | C | A | 0.562 | Schizophrenia | TRUE | reported | igd | 0.000568 | 74.30762 |
| 8 | 130644 | 1.21E-08 | 17070926 | 0.0098 | 0.055999 | ieu-b-5102 | rs4921741 | G | A | 0.261 | Schizophrenia | TRUE | reported | igd | 0.00025 | 32.65151 |
| 8 | 130644 | 3.48E-08 | 65605878 | 0.0086 | 0.047595 | ieu-b-5102 | rs10957321 | A | G | 0.511 | Schizophrenia | TRUE | reported | igd | 0.000234 | 30.6279 |
| 8 | 130644 | 5.07E-10 | 4180090 | 0.0108 | 0.067305 | ieu-b-5102 | rs10103330 | A | T | 0.194 | Schizophrenia | TRUE | reported | igd | 0.000297 | 38.83654 |
| 8 | 130644 | 4.97E-10 | 111580570 | 0.0116 | 0.072205 | ieu-b-5102 | rs10086619 | G | A | 0.161 | Schizophrenia | TRUE | reported | igd | 0.000296 | 38.74488 |
| 9 | 130644 | 4.87E-09 | 77358745 | 0.0088 | -0.0517 | ieu-b-5102 | rs500102 | C | T | 0.591 | Schizophrenia | TRUE | reported | igd | 0.000264 | 34.51536 |
| 9 | 130644 | 1.25E-08 | 36319928 | 0.0088 | 0.050399 | ieu-b-5102 | rs2381411 | C | T | 0.402 | Schizophrenia | TRUE | reported | igd | 0.000251 | 32.79985 |
| 9 | 130644 | 2.11E-09 | 14509105 | 0.0121 | 0.072495 | ieu-b-5102 | rs498591 | T | A | 0.146 | Schizophrenia | TRUE | reported | igd | 0.000275 | 35.89579 |
| 9 | 130644 | 2.94E-08 | 138378856 | 0.0126 | -0.0696 | ieu-b-5102 | rs2078266 | G | A | 0.829 | Schizophrenia | TRUE | reported | igd | 0.000234 | 30.51262 |
| 9 | 130644 | 5.80E-10 | 22767164 | 0.0086 | 0.053496 | ieu-b-5102 | rs505061 | A | C | 0.488 | Schizophrenia | TRUE | reported | igd | 0.000296 | 38.69316 |
| 9 | 130644 | 2.54E-08 | 101071522 | 0.0118 | 0.065595 | ieu-b-5102 | rs3824451 | C | T | 0.153 | Schizophrenia | TRUE | reported | igd | 0.000236 | 30.90097 |
| 10 | 130644 | 3.33E-12 | 18726326 | 0.0142 | 0.098904 | ieu-b-5102 | rs6482437 | C | A | 0.888 | Schizophrenia | TRUE | reported | igd | 0.000371 | 48.51107 |
| 10 | 130644 | 4.44E-09 | 92789488 | 0.0102 | -0.0601 | ieu-b-5102 | rs61857878 | T | A | 0.254 | Schizophrenia | TRUE | reported | igd | 0.000266 | 34.71398 |
| 10 | 130644 | 4.37E-09 | 3821561 | 0.0089 | 0.052399 | ieu-b-5102 | rs17731 | A | G | 0.365 | Schizophrenia | TRUE | reported | igd | 0.000265 | 34.66272 |
| 10 | 130644 | 1.94E-08 | 54063083 | 0.0093 | -0.0524 | ieu-b-5102 | rs12771371 | A | G | 0.311 | Schizophrenia | TRUE | reported | igd | 0.000243 | 31.74935 |
| 10 | 130644 | 1.77E-17 | 104906211 | 0.0155 | -0.1317 | ieu-b-5102 | rs11191580 | C | T | 0.09 | Schizophrenia | TRUE | reported | igd | 0.000552 | 72.19736 |
| 11 | 130644 | 1.35E-14 | 113392994 | 0.0092 | -0.0705 | ieu-b-5102 | rs2514218 | T | C | 0.344 | Schizophrenia | TRUE | reported | igd | 0.000449 | 58.71417 |
| 11 | 130644 | 2.39E-08 | 81178838 | 0.0096 | 0.053496 | ieu-b-5102 | rs72943392 | C | G | 0.289 | Schizophrenia | TRUE | reported | igd | 0.000238 | 31.05193 |
| 11 | 130644 | 2.80E-08 | 134247187 | 0.0094 | -0.0523 | ieu-b-5102 | rs7113199 | C | A | 0.704 | Schizophrenia | TRUE | reported | igd | 0.000237 | 30.95372 |
| 11 | 130644 | 6.56E-09 | 57585662 | 0.0091 | 0.0528 | ieu-b-5102 | rs708228 | T | C | 0.329 | Schizophrenia | TRUE | reported | igd | 0.000258 | 33.6646 |
| 11 | 130644 | 1.52E-09 | 130805334 | 0.0086 | -0.0522 | ieu-b-5102 | rs7112616 | C | T | 0.498 | Schizophrenia | TRUE | reported | igd | 0.000282 | 36.84631 |
| 11 | 130644 | 9.58E-12 | 133827733 | 0.0108 | -0.0736 | ieu-b-5102 | rs3802924 | C | A | 0.212 | Schizophrenia | TRUE | reported | igd | 0.000355 | 46.44553 |
| 11 | 130644 | 2.40E-09 | 24389235 | 0.0086 | 0.051504 | ieu-b-5102 | rs11027839 | C | A | 0.5 | Schizophrenia | TRUE | reported | igd | 0.000274 | 35.86534 |
| 11 | 130644 | 4.89E-08 | 28642653 | 0.0089 | -0.0483 | ieu-b-5102 | rs4636654 | A | G | 0.402 | Schizophrenia | TRUE | reported | igd | 0.000225 | 29.45676 |
| 11 | 130644 | 1.05E-14 | 46343189 | 0.011 | 0.084905 | ieu-b-5102 | rs12285419 | A | C | 0.188 | Schizophrenia | TRUE | reported | igd | 0.000456 | 59.57573 |
| 11 | 130644 | 1.56E-14 | 124612932 | 0.0092 | -0.0705 | ieu-b-5102 | rs12293670 | G | A | 0.34 | Schizophrenia | TRUE | reported | igd | 0.000449 | 58.71417 |
| 12 | 130644 | 1.04E-21 | 2413803 | 0.0087 | -0.0838 | ieu-b-5102 | rs1860002 | T | C | 0.543 | Schizophrenia | TRUE | reported | igd | 0.00071 | 92.77467 |
| 12 | 130644 | 1.59E-08 | 104631552 | 0.0098 | 0.055502 | ieu-b-5102 | rs10861176 | A | G | 0.737 | Schizophrenia | TRUE | reported | igd | 0.000245 | 32.07451 |
| 12 | 130644 | 3.93E-17 | 110723245 | 0.0089 | 0.075004 | ieu-b-5102 | rs4766428 | T | C | 0.441 | Schizophrenia | TRUE | reported | igd | 0.000543 | 71.01987 |
| 12 | 130644 | 1.59E-09 | 72259954 | 0.0145 | 0.087499 | ieu-b-5102 | rs12303743 | C | G | 0.094 | Schizophrenia | TRUE | reported | igd | 0.000279 | 36.41343 |
| 12 | 130644 | 4.92E-14 | 123650335 | 0.0098 | -0.0736 | ieu-b-5102 | rs1615350 | T | C | 0.744 | Schizophrenia | TRUE | reported | igd | 0.000432 | 56.40782 |
| 12 | 130644 | 2.77E-08 | 124476873 | 0.009 | 0.050199 | ieu-b-5102 | rs12833624 | T | C | 0.342 | Schizophrenia | TRUE | reported | igd | 0.000238 | 31.11014 |
| 12 | 130644 | 1.03E-08 | 53760710 | 0.0094 | 0.054002 | ieu-b-5102 | rs10876446 | C | G | 0.312 | Schizophrenia | TRUE | reported | igd | 0.000253 | 33.00354 |
| 12 | 130644 | 1.15E-15 | 57682956 | 0.0162 | -0.1301 | ieu-b-5102 | rs61937595 | T | C | 0.092 | Schizophrenia | TRUE | reported | igd | 0.000493 | 64.49181 |
| 12 | 130644 | 4.43E-11 | 95195293 | 0.0086 | -0.0568 | ieu-b-5102 | rs6538539 | T | G | 0.549 | Schizophrenia | TRUE | reported | igd | 0.000334 | 43.61476 |
| 13 | 130644 | 1.69E-08 | 38860697 | 0.0088 | 0.049495 | ieu-b-5102 | rs2455415 | T | C | 0.413 | Schizophrenia | TRUE | reported | igd | 0.000242 | 31.63362 |
| 13 | 130644 | 1.80E-09 | 74325499 | 0.0099 | 0.059601 | ieu-b-5102 | rs12877581 | C | G | 0.277 | Schizophrenia | TRUE | reported | igd | 0.000277 | 36.24398 |
| 13 | 130644 | 4.35E-12 | 79930079 | 0.0088 | -0.0612 | ieu-b-5102 | rs9318627 | C | A | 0.4 | Schizophrenia | TRUE | reported | igd | 0.00037 | 48.36275 |
| 14 | 130644 | 3.88E-14 | 72417326 | 0.0099 | -0.0751 | ieu-b-5102 | rs2332700 | G | C | 0.753 | Schizophrenia | TRUE | reported | igd | 0.00044 | 57.54161 |
| 14 | 130644 | 3.01E-13 | 104255569 | 0.0091 | 0.066503 | ieu-b-5102 | rs10873538 | G | T | 0.334 | Schizophrenia | TRUE | reported | igd | 0.000409 | 53.40653 |
| 14 | 130644 | 2.21E-08 | 30300361 | 0.0089 | 0.049905 | ieu-b-5102 | rs1953205 | A | T | 0.485 | Schizophrenia | TRUE | reported | igd | 0.000241 | 31.44112 |
| 14 | 130644 | 1.86E-12 | 33303540 | 0.0087 | 0.061301 | ieu-b-5102 | rs12883788 | T | C | 0.458 | Schizophrenia | TRUE | reported | igd | 0.00038 | 49.64681 |
| 14 | 130644 | 3.05E-08 | 51655145 | 0.0094 | 0.051799 | ieu-b-5102 | rs2999392 | T | C | 0.691 | Schizophrenia | TRUE | reported | igd | 0.000232 | 30.36515 |
| 14 | 130644 | 2.21E-09 | 99733384 | 0.0093 | -0.0557 | ieu-b-5102 | rs1540840 | C | G | 0.475 | Schizophrenia | TRUE | reported | igd | 0.000274 | 35.87002 |
| 15 | 130644 | 1.01E-10 | 40567237 | 0.0097 | 0.063004 | ieu-b-5102 | rs56205728 | A | G | 0.287 | Schizophrenia | TRUE | reported | igd | 0.000323 | 42.18733 |
| 15 | 130644 | 7.27E-11 | 83368738 | 0.0089 | -0.058 | ieu-b-5102 | rs4779050 | G | T | 0.633 | Schizophrenia | TRUE | reported | igd | 0.000325 | 42.46185 |
| 15 | 130644 | 1.94E-09 | 44080737 | 0.0097 | 0.058403 | ieu-b-5102 | rs62018952 | C | T | 0.726 | Schizophrenia | TRUE | reported | igd | 0.000277 | 36.25064 |
| 15 | 130644 | 2.21E-13 | 61872197 | 0.0087 | 0.063504 | ieu-b-5102 | rs35351411 | C | A | 0.544 | Schizophrenia | TRUE | reported | igd | 0.000408 | 53.27979 |
| 15 | 130644 | 1.13E-15 | 78868398 | 0.0102 | -0.0816 | ieu-b-5102 | rs2456020 | T | C | 0.241 | Schizophrenia | TRUE | reported | igd | 0.00049 | 63.99651 |
| 15 | 130644 | 2.79E-21 | 91426560 | 0.0089 | -0.0843 | ieu-b-5102 | rs4702 | A | G | 0.56 | Schizophrenia | TRUE | reported | igd | 0.000686 | 89.72507 |
| 16 | 130644 | 1.58E-14 | 29994922 | 0.0087 | -0.0671 | ieu-b-5102 | rs3814883 | T | C | 0.467 | Schizophrenia | TRUE | reported | igd | 0.000455 | 59.47988 |
| 16 | 130644 | 3.00E-08 | 58659307 | 0.0087 | -0.0482 | ieu-b-5102 | rs149165 | G | T | 0.446 | Schizophrenia | TRUE | reported | igd | 0.000235 | 30.69304 |
| 16 | 130644 | 5.77E-09 | 89559297 | 0.0096 | 0.055798 | ieu-b-5102 | rs4575535 | G | A | 0.704 | Schizophrenia | TRUE | reported | igd | 0.000259 | 33.78246 |
| 16 | 130644 | 5.69E-11 | 13753384 | 0.0101 | 0.066503 | ieu-b-5102 | rs8055219 | A | G | 0.229 | Schizophrenia | TRUE | reported | igd | 0.000332 | 43.35452 |
| 17 | 130644 | 5.48E-10 | 12875908 | 0.0109 | 0.067605 | ieu-b-5102 | rs73292401 | A | T | 0.192 | Schizophrenia | TRUE | reported | igd | 0.000294 | 38.46729 |
| 17 | 130644 | 1.99E-09 | 19141582 | 0.0139 | -0.0831 | ieu-b-5102 | rs57433322 | G | C | 0.122 | Schizophrenia | TRUE | reported | igd | 0.000274 | 35.74058 |
| 17 | 130644 | 2.64E-11 | 44289832 | 0.0092 | -0.0612 | ieu-b-5102 | rs2696466 | G | A | 0.403 | Schizophrenia | TRUE | reported | igd | 0.000339 | 44.24872 |
| 18 | 130644 | 8.94E-13 | 77578986 | 0.0108 | 0.0774 | ieu-b-5102 | rs11664298 | A | G | 0.193 | Schizophrenia | TRUE | reported | igd | 0.000393 | 51.35966 |
| 18 | 130644 | 1.59E-08 | 27500959 | 0.01 | -0.0567 | ieu-b-5102 | rs9304548 | A | C | 0.748 | Schizophrenia | TRUE | reported | igd | 0.000246 | 32.15022 |
| 18 | 130644 | 4.59E-08 | 50746748 | 0.0086 | 0.047196 | ieu-b-5102 | rs4632195 | T | C | 0.52 | Schizophrenia | TRUE | reported | igd | 0.00023 | 30.11717 |
| 18 | 130644 | 5.11E-16 | 53200117 | 0.0086 | 0.069897 | ieu-b-5102 | rs9636107 | G | A | 0.471 | Schizophrenia | TRUE | reported | igd | 0.000505 | 66.056 |
| 19 | 130644 | 3.59E-10 | 11849736 | 0.0179 | 0.112296 | ieu-b-5102 | rs72986630 | T | C | 0.068 | Schizophrenia | TRUE | reported | igd | 0.000301 | 39.35645 |
| 19 | 130644 | 8.29E-12 | 50162909 | 0.0094 | -0.0641 | ieu-b-5102 | rs7251 | G | C | 0.328 | Schizophrenia | TRUE | reported | igd | 0.000356 | 46.50139 |
| 19 | 130644 | 2.80E-16 | 19518316 | 0.0089 | 0.073205 | ieu-b-5102 | rs1000237 | A | T | 0.355 | Schizophrenia | TRUE | reported | igd | 0.000518 | 67.65476 |
| 19 | 130644 | 3.01E-09 | 30987423 | 0.0101 | 0.059899 | ieu-b-5102 | rs2053079 | G | A | 0.237 | Schizophrenia | TRUE | reported | igd | 0.000269 | 35.17094 |
| 20 | 130644 | 8.96E-16 | 37485458 | 0.0089 | 0.071904 | ieu-b-5102 | rs4812325 | A | G | 0.616 | Schizophrenia | TRUE | reported | igd | 0.000499 | 65.27124 |
| 20 | 130644 | 7.26E-09 | 48105317 | 0.011 | 0.063696 | ieu-b-5102 | rs11696755 | C | T | 0.184 | Schizophrenia | TRUE | reported | igd | 0.000257 | 33.53011 |
| 20 | 130644 | 2.86E-08 | 62150128 | 0.0202 | 0.112296 | ieu-b-5102 | rs113264400 | C | T | 0.048 | Schizophrenia | TRUE | reported | igd | 0.000237 | 30.90432 |
| 22 | 126538 | 3.70E-08 | 39247750 | 0.0348 | 0.1915 | ieu-b-5102 | rs6001259 | T | C | 0.016 | Schizophrenia | TRUE | reported | igd | 0.000239 | 30.28114 |
| 22 | 130644 | 3.26E-09 | 39988175 | 0.0086 | -0.051 | ieu-b-5102 | rs132582 | T | C | 0.545 | Schizophrenia | TRUE | reported | igd | 0.000269 | 35.16326 |
| 22 | 130644 | 3.00E-14 | 42370991 | 0.0086 | 0.065595 | ieu-b-5102 | rs5751191 | C | T | 0.501 | Schizophrenia | TRUE | reported | igd | 0.000445 | 58.17538 |
| 22 | 130644 | 4.46E-08 | 50290678 | 0.0106 | 0.058095 | ieu-b-5102 | rs8138941 | A | G | 0.204 | Schizophrenia | TRUE | reported | igd | 0.00023 | 30.03749 |
| 22 | 130263 | 7.44E-09 | 51103091 | 0.0095 | 0.0549 | ieu-b-5102 | rs6010045 | C | T | 0.698 | Schizophrenia | TRUE | reported | igd | 0.000256 | 33.39548 |
| 2 | 117706 | 2.38E-06 | 72150177 | 0.007746 | 0.03655 | ukb-d-20544_15 | rs139059714 | T | A | 0.009324 | Anxiety | TRUE | reported | igd | 0.000189 | 22.26674 |
| 3 | 117706 | 1.29E-06 | 95114857 | 0.006583 | 0.031868 | ukb-d-20544_15 | rs141793750 | A | C | 0.012815 | Anxiety | TRUE | reported | igd | 0.000199 | 23.4357 |
| 3 | 117706 | 2.58E-06 | 134107044 | 0.001815 | 0.008533 | ukb-d-20544_15 | rs55792881 | G | C | 0.20203 | Anxiety | TRUE | reported | igd | 0.000188 | 22.11034 |
| 3 | 117706 | 3.81E-06 | 48649457 | 0.001911 | -0.00883 | ukb-d-20544_15 | rs1264191 | C | T | 0.822246 | Anxiety | TRUE | reported | igd | 0.000181 | 21.35832 |
| 3 | 117706 | 2.63E-06 | 85033203 | 0.001623 | -0.00762 | ukb-d-20544_15 | rs1694895 | T | C | 0.355039 | Anxiety | TRUE | reported | igd | 0.000187 | 22.07338 |
| 4 | 117706 | 4.05E-06 | 32953162 | 0.00182 | 0.00839 | ukb-d-20544_15 | rs73212997 | C | A | 0.196882 | Anxiety | TRUE | reported | igd | 0.00018 | 21.24481 |
| 5 | 117706 | 3.06E-06 | 122039997 | 0.001458 | -0.0068 | ukb-d-20544_15 | rs17164793 | A | G | 0.529742 | Anxiety | TRUE | reported | igd | 0.000185 | 21.77907 |
| 6 | 117706 | 4.20E-06 | 31672268 | 0.003082 | 0.014183 | ukb-d-20544_15 | rs34406901 | G | A | 0.056803 | Anxiety | TRUE | reported | igd | 0.00018 | 21.17103 |
| 7 | 117706 | 2.02E-06 | 89029584 | 0.003063 | -0.01455 | ukb-d-20544_15 | rs17169480 | C | T | 0.060762 | Anxiety | TRUE | reported | igd | 0.000192 | 22.57891 |
| 7 | 117706 | 2.76E-06 | 12250378 | 0.001456 | 0.006825 | ukb-d-20544_15 | rs3807866 | A | G | 0.408921 | Anxiety | TRUE | reported | igd | 0.000187 | 21.97894 |
| 9 | 117706 | 1.66E-06 | 90799436 | 0.006994 | 0.03351 | ukb-d-20544_15 | rs79866996 | G | A | 0.012603 | Anxiety | TRUE | reported | igd | 0.000195 | 22.95723 |
| 9 | 117706 | 8.74E-09 | 11724099 | 0.001724 | -0.00992 | ukb-d-20544_15 | rs62555663 | A | C | 0.222075 | Anxiety | TRUE | reported | igd | 0.000281 | 33.10677 |
| 10 | 117706 | 1.45E-06 | 13835250 | 0.001516 | -0.0073 | ukb-d-20544_15 | rs10906487 | C | T | 0.343945 | Anxiety | TRUE | reported | igd | 0.000197 | 23.21515 |
| 11 | 117706 | 1.73E-06 | 65681104 | 0.002721 | 0.013017 | ukb-d-20544_15 | rs141172478 | T | C | 0.079202 | Anxiety | TRUE | reported | igd | 0.000194 | 22.87996 |
| 12 | 117706 | 2.48E-06 | 107822500 | 0.003606 | -0.01698 | ukb-d-20544_15 | rs78308659 | C | T | 0.041272 | Anxiety | TRUE | reported | igd | 0.000188 | 22.18103 |
| 17 | 117706 | 4.93E-06 | 15175910 | 0.002676 | 0.012222 | ukb-d-20544_15 | rs117713898 | T | C | 0.08071 | Anxiety | TRUE | reported | igd | 0.000177 | 20.86653 |
| 22 | 117706 | 4.95E-06 | 46114797 | 0.004724 | 0.021577 | ukb-d-20544_15 | rs146972845 | A | G | 0.026724 | Anxiety | TRUE | reported | igd | 0.000177 | 20.85994 |
